# Hosts and Commensal Bacteria Synergistically Antagonize Opportunistic Pathogens at the Single‐Cell Resolution

**DOI:** 10.1002/advs.202500582

**Published:** 2025-05-23

**Authors:** Sheng Zhang, Ziguang Wang, Anqi Liu, Jinshu Li, Jingjing Zhuang, Xiaowen Ji, Paul I. Mulama, Maoye Li, Haiqun Cao, Eng‐King Tan, Wei Liu

**Affiliations:** ^1^ School of Plant Protection Anhui Agricultural University Anhui Province Key Laboratory of Crop Integrated Pest Management Anhui Province Key Laboratory of Resource Insect Biology and Innovative Utilization Hefei 230036 China; ^2^ College of Life Sciences Nankai University Tianjin 300071 China; ^3^ Department of Neurology National Neuroscience Institute Singapore 308433 Singapore

**Keywords:** bacterial single‐cell RNA‐seq, heterogeneity, metabolic adaptations, pathogenicity

## Abstract

Natural microbes coexist in a diverse species population with competition for space and nutrient resources. However, the molecular mechanisms underpinning the regulatory networks of microbes among themselves and with their host are still in infancy. Here, it is reported that *Drosophila* and the commensal *Lactiplantibacillus plantarum* form an alliance to compete with the pathogenic *Serratia marcescens* using the integrated three‐species model system. In the dual‐species model, larvae diminish the *L. plantarum* population, but reversibly increase lactate production through altering its transcriptional reprogramming. In the tripartite‐species model, larvae facilitate the growth of *L. plantarum* that confers colonization resistance against *S. marcescens*. On the other hand, *S. marcescens* launches sophisticated arms race strategies to impair colonization resistance by sensing lactate derived from *L. plantarum*. More importantly, the *S. marcescens* population challenged with *Drosophila* and *L. plantarum* adaptively diverge into virulent and reduced virulence subclusters with an increase in resistance heterogeneity. To form the alliance with *Drosophila*, heterogeneity in lactate generation is broadened among *L. plantarum* subpopulations. Altogether, these findings provide an insight into the host‐commensal‐pathogen symbiosis at both bulk and single‐cell resolutions, advancing fundamental concepts of precise manipulation of bacterial communities.

## Graphical abstract

In the tripartite‐species model, *Drosophila* larvae stimulate lactate production through excreting digestion enzymes. *S. marcescens* population senses a cue of lactate and tends to diverge into virulent and reduced virulence subclusters with remarkable heterogeneity to metabolize carbon metabolism and counter colonization resistance, giving a fitness advantage in a niche.

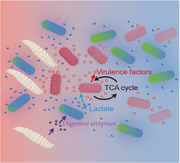

## 1 Introduction

Massive communities of diverse symbiotic microbes colonize an individual's body and surroundings throughout their lifetime, exerting profound effects on aspects of host physiopathology, including development, metabolism, behaviors and diseases.^[^ ^1^, ^2^, ^3^ ^]^ Microbes that rely on the relationship with their hosts can be categorized as mutualists, pathogens, or commensals.^[^ ^4^ ^]^ Lines of evidence reveal that the microbiome provides a routine barrier to resist pathogen invasion and colonization through nutrient depletion, niche occupation, and immunomodulation, a phenomenon known as colonization resistance.^[^ ^5^, ^6^ ^]^ Conversely, pathogens engage in an evolutionary arms race to overcome colonization resistance.^[^ ^7^ ^]^ The long‐term coexistence of two antagonistic populations generally remains stable inside/around the host, but the molecular mechanisms underpinning the interactions of microbes among themselves and with their host remain elusive. While interspecies interactions are enormously influenced by microbiota compositions,^[^ ^8^, ^9^ ^]^ environmental fluctuations usually happen at much shorter time scales, rendering genetic mutations or species displacement improbable.^[^ ^10^, ^11^ ^]^ Instead, microbes swiftly adapt to ambient changes by launching sophisticated transcriptional reprogramming.^[^ ^12^, ^13^ ^]^ The microbiome is thus being conceptualized as “an ecosystem on a leash”, where transcriptional profiles are robustly influenced by host control and new strain immigration.^[^ ^14^ ^]^ In this regard, a major challenge in interpreting microbiome signatures is to advance beyond descriptive composition‐level profiling towards disentangling the transcriptional interactome among microbes, hosts, and pathogens.^[^ ^15^ ^]^

Pathogens have evolved a panoply of defense strategies to evade colonization resistance.^[^ ^9^, ^16^ ^]^ Successful colonization depends primarily on the precise regulation of virulence gene expression during different phases of infection to outcompete commensal rivals.^[^ ^9^, ^17^ ^]^ If the pathogen fails to express virulence factors rapidly enough, it could be eradicated by the microbiome before achieving its optimal virulence.^[^ ^18^, ^19^ ^]^ However, the expression of these genes is energetically costly, so pathogens adopt bistable resistance regulation, resulting in phenotypic heterogeneity within the population.^[^ ^20^ ^]^ In this context, pathogens express virulence factors in a bistable manner, leading to a slow‐growing, virulent subpopulation and a fast‐growing, reduced virulence subpopulation. It has been thought that phenotypic heterogeneity can accelerate the rate of adaptive evolution in bacterial populations encountering extreme environmental risks by rapidly generating a subpopulation with novel phenotypic traits.^[^ ^21^ ^]^ Recent studies find that bacteria can stochastically differentiate into subpopulations with phenotypic heterogeneity even under homogeneous conditions, to hedge the risk of their extinction.^[^ ^22^, ^23^, ^24^ ^]^ However, traditional bulk RNA‐seq techniques merely profile the average gene expression of a population of cells, masking cell‐to‐cell variations that are hidden within microbial population behavior. Fortunately, the advent of bacterial single‐cell RNA sequencing (scRNA‐seq) is revolutionizing the analysis of phenotypic heterogeneity of individual cells,^[^ ^25^, ^26^, ^27^ ^]^ paving the way for new strategies to combat pathogens. For instance, this approach facilitates the identification of persister cells across multiple genotypes that are in a transitional phase between stationary and exponential growth, thereby advancing the understanding of bacterial pathogenesis.^[^ ^27^, ^28^ ^]^

Constituent species diversity and spatiotemporally transcriptional dynamics make colonization resistance a challenging phenotype in mammals. In contrast, *D. melanogaster* harbors a consortium of culturable species with low diversity, allowing us to systematically dissect the interactome of multiple species.^[^ ^2^, ^29^ ^]^ In the wild, saprophagous *Drosophila* larvae devour rotten fruits that are persistently colonized with diverse commensals as well as pathogens.^[^ ^30^ ^]^ Distinguishing from internal growth in mammals, *Drosophila* repopulate its microbiome through the frequent ingestion of bacteria from environmental sources, establishing an open symbiosis.^[^ ^30^, ^31^, ^32^ ^]^ As a result, both microbial quantity and quality in such an open system would exert a more profound effect on aspects of *Drosophila* life than in a closed system.^[^ ^31^ ^]^ However, many of the underlying mechanisms and principles are universally applicable, spanning vertebrates, invertebrates, plants, and even some single‐celled hosts.^[^ ^14^, ^33^ ^]^ In the present study, bulk and single‐cell RNA‐seq techniques were applied to explore the phenotypic heterogeneity of *Drosophila* commensals and pathogens within an integrated *Drosophila*‐commensal‐pathogen model system, highlighting the intricacy of tripartite relationships in an ecological system.

## 2 Results

### 2.1 *L. plantarum* Undergoes Transcriptomic Shifts in the Dual‐Species Interaction

**Figure 1:**
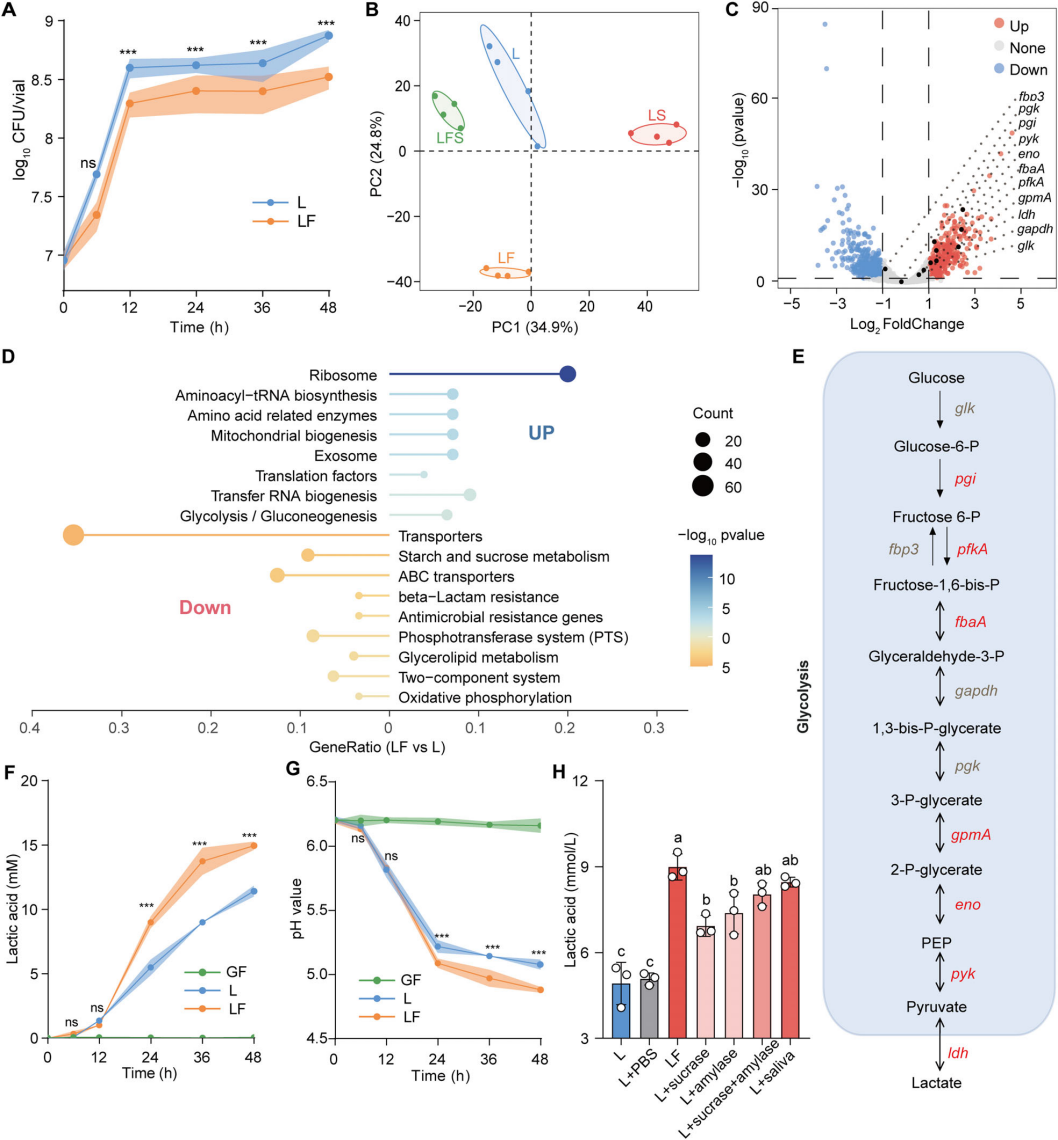
*Drosophila* larvae stimulate the lactate production of *L. plantarum*. A) The time‐course load of *L. plantarum* cultured alone (L) and co‐cultured with 40 larvae (LF). (*n*   =  9). The solid line and shaded area of loading curves show mean ± SD, and the significance analysis was performed by unpaired two‐sided Student's *t*‐test. ns (non‐significance), *p*  >  0.05; ***p  <  0.001. B) Principal component analysis (PCA) of *L. plantarum* gene expression at 24 h timepoint after inoculation in L, LF, LS (*L. plantarum*/*S. marcescens*), and LFS (*L. plantarum*/fly/*S. marcescens*) groups (*n*   =   4). C) Volcano plots showing differentially regulated genes of *L. plantarum* between L and LF groups, based on the criteria of a Log_2_fold change of > 1 or <  −1 and a Bonferroni‐corrected two‐tailed moderated *t*‐test with *p* < 0.05 (dark blue and dark red). D) Kyoto Encyclopedia of Genes and Genomes (KEGG) enrichment analysis of the significantly upregulated (log_2_fold > 1; *p* < 0.05) and downregulated (log_2_fold < ‐1; *p* < 0.05) genes in *L. plantarum* within LF group compared to S group. E) The diagram of the glucose catabolism pathway of *L. plantarum*. Glucose is catabolized to pyruvate that can be finally reduced to lactate. The genes coding for glycolytic enzymes upregulated are shown in red. F,G) The curves of F) lactate levels and G) pH values over time in GF (40 germ‐free fly larvae), L, and LF groups. (*n*  =   3). The solid line and shaded area of curves show mean ± SD, and the significance analysis between L and LF was performed by unpaired two‐sided Student's *t*‐test. ns (non‐significance), *p*  >  0.05; ***p  <  0.001. H) The lactate levels in the medium were measured after 24 h of incubation, supplemented with either PBS, sucrase, amylase, or saliva (*n* = 3). The data represent the means ± SD, and the different letters above the columns in E and F denote statistically significant differences between groups (*p*  < 0.05). One‐way ANOVA followed by Tukey's test for multiple comparisons.

Our previous results showed that *Drosophila* larvae suppressed the overgrowth and pathogenicity of *S. marcescens* in the shared niche.^[^ ^34^ ^]^ To study the potential effect of the host on commensal bacteria, we employed *Drosophila* larvae mono‐associated with *L. plantarum* as depicted (Figure S1A, Supporting Information). Similarly, larval colonization diminished *L. plantarum* burdens in the habitat (LF, *L. plantarum*/fly) over time compared to *L. plantarum* alone (L, single *L. plantarum*; **Figure** **1**A; Figure S1B, Supporting Information), suggesting that the host still exerts control over its commensals. This inhibitory effect is likely attributable to host factors rather than food scarcity, since fresh food was continuously provided through their burrowing.^[^ ^34^, ^35^ ^]^ To understand gene expression regulation of commensals in adaptation to changing environments, bulk RNA sequencing was performed on the *L. plantarum* population in L and LF groups. A principal component analysis (PCA) showed a clear separation between L and LF groups (Figure 1B). Cocultured *L. plantarum* exhibited 336 gene upregulation and 395 gene downregulation compared to single *L. plantarum* (Figure 1C). The functions of the differentially expressed genes (DEGs) were assigned using a KEGG analysis. Pathway enrichment analysis generally revealed that transcripts associated with ribosome, aminoacyl‐tRNA biosynthesis, translation factors, transporters, antimicrobial resistance genes, and two‐component system were significantly altered in the presence of larvae (Figure 1D), suggesting that *L. plantarum* undergoes substantial transcriptional reprogramming in adaptation to environmental changes caused by the host.

Among these enriched pathways, an upregulated pathway of glycolysis in the LF group caught our attention, because most key genes were differentially expressed (Figure 1E; Figure S1C, Supporting Information). To verify it, the expression levels of glycolysis‐related genes were examined by qRT‐PCR. Indeed, *L. plantarum* exhibited 1.37‐ to 3.38‐fold higher transcriptional levels of *pgi*, *pfka*, and *gapdh* when cocultured with larvae than when alone (Figure S1D, Supporting Information). Moreover, cocultured *L. plantarum* generated approximately 1.5‐fold higher levels of lactate, the end product of glycolysis, than single *L. plantarum* (Figure 1F; Figure S1E, Supporting Information). To rule out the possibility that the elevated lactate was produced by the larvae, we measured lactate levels in a diet containing the same number of germ‐free (GF) larvae alone. No significant increase in lactate content was observed over time or with an increasing number of larvae (Figure S1F, Supporting Information). These findings suggest that lactate accumulation is primarily attributed to the metabolism of *L. plantarum* rather than the larvae. Correspondingly, pH values of the medium were lower in the LF group than in the L group (Figure 1G; Figure S1G, Supporting Information), as lactate is a short‐chain fatty acid with a p*K*a of 3.86. Consistently, the pH values remained stable over time or with an increasing number of larvae (Figure S1H, Supporting Information). Combined with the lower bacterial load in the LF group (Figure 1A), we thereby deterred that the host‐triggered lactate production activity of *L. plantarum* overwhelmed the population size of *L. plantarum* alone. Larvae egest digestion enzymes, like amylase and sucrase, to perform external digestion of their food,^[^ ^30^, ^36^ ^]^ which prompted us to test whether saliva or digestive enzymes could potentially contribute to the increase in lactate in the medium. Indeed, the addition of larval saliva significantly enhanced lactate contents in the medium (Figure 1H), while it simultaneously reduced *L. plantarum* loads (Figure S1I, Supporting Information). Furthermore, both amylase and sucrase significantly increased lactate production in the medium, although neither enzyme affected *L. plantarum* burdens in the food. Taken together, these results indicate that larvae promote lactate generation of *L. plantarum* through excreting digestive enzymes.

### 2.2 Tripartite *Drosophila*‐*L. plantarum*–*S. marcescens* Interactions

**Figure 2:**
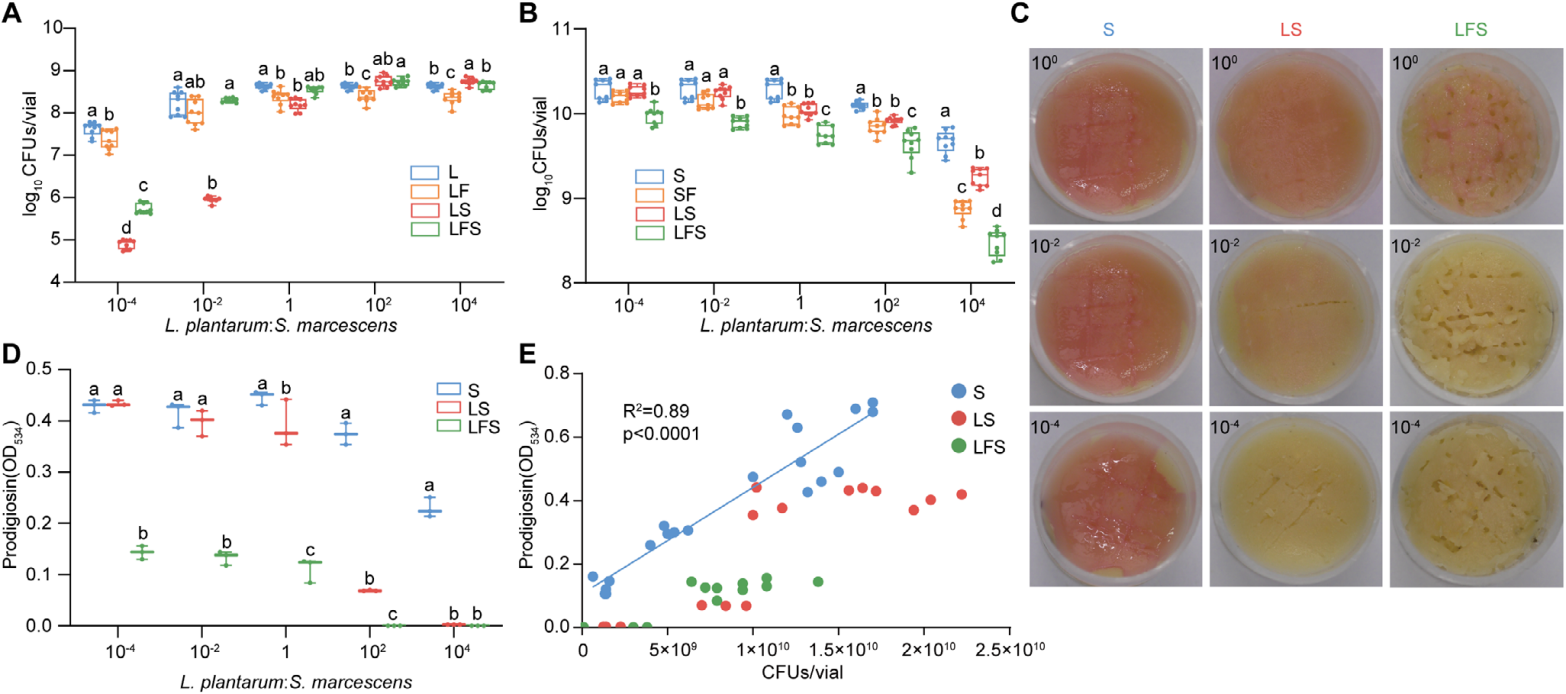
*Drosophila* larvae regulate the population sizes of *L. plantarum* and *S. marcescens*. A) The 24 h timepoint load of *L. plantarum* in L, LF, cocultured with the *S. marcescens* (LS), and LFS groups was measured, with approximately 10⁷ cells inoculated at different initial ratios of *L. plantarum* to *S. marcescens* (1:10⁻⁴, 1:10⁻^2^, 100:1, 10⁻^2^:1, 10⁻⁴:1; *n* = 9). B) The 24 h timepoint load of *S. marcescens* in *S. marcescens* alone (S), co‐cultured with 40 larvae (SF), co‐cultured with the *L. plantarum* (LS), and LFS with different initial inoculation ratios of *L. plantarum*: *S. marcescens* (1:10^−4^, 1:10^−2^, 10^0^:1, 10^−2^:1, 10^−4^:1; *n*  =   9). C) Representative images of the surface slick with different initial inoculation ratios in the S, LS and SFL groups. D) Prodigiosin production of *S. marcescens* at 24 h timepoint after inoculation under different initial inoculation ratios (*n*   =  3). Prodigiosin production was assessed with the spectrometer. E) The regression line for the S group was higher than the point for the LS and SFL groups. The different letters above the columns in A, B and D denote statistically significant differences between groups (*p* < 0.05). Two‐way ANOVA followed by Tukey's test for multiple comparisons.

To address tripartite interactions, we employed a more complex system containing *Drosophila*, *L. plantarum*, and an opportunistic pathogen *S. marcescens*,^[^ ^10^, ^37^ ^]^ providing more realistic conditions with competitive and cooperative interactions between bacteria and hosts. Our result showed that *L. plantarum* at low initial inoculation ratios of *L. plantarum* to *S. marcescens* reached higher population sizes in the presence of larvae (LFS, *L. plantarum*/fly/*S. marcescens*) than in the absence of larvae (LS, *L. plantarum*/*S. marcescens*; **Figure** **2**A; Figure S2A, Supporting Information). In addition, the loads of *L. plantarum* at high inoculation ratios of *L. plantarum* to *S. marcescens* were also higher in the LFS group than in the LF group. These findings suggest that the fly alters the environment to discriminatively facilitate the growth of commensals in the context of pathogens. Contrastingly, larvae exacerbated a decline in *S. marcescens* loads caused by *L. plantarum* (Figure 2B; Figure S2B, Supporting Information), indicating that the host and commensals coordinate to antagonize pathogens. As expected, *S. marcescens* loads were significantly higher in the SF group than in the LSF group, ruling out the possibility that larvae alone are sufficient to achieve robust inhibition. Notably, *S. marcescens* exhibited a faster growth rate than *L. plantarum*, and still reached approximately half of the total bacteria (≈5×10^8^) at 24‐timepoint following inoculation with the 10^4^: 1 ratio of *L. plantarum* to *S. marcescens*, mimicking the invasion and colonization of a pathogen with predominant commensals in the niche.

### 2.3 *S. marcescens* Performs Global Transcriptional Adaptation

We firstly assessed a visibly reliable bioindicator prodigiosin to evaluate the overall metabolic activities of *S. marcescens*.^[^ ^38^, ^39^, ^40^ ^]^ We observed a plummet in prodigiosin production in *S. marcescens* when inflicted with a couple of *L. plantarum* and larvae (Figure 2C,D). To rule out the possibility that the reduction in prodigiosin production resulted from lower *S. marcescens* burdens, we calculated a regression line of prodigiosin levels with numbers of bacterial cells. The total spots of the LFS group deviated far below the linear standard curve (Figure 2E), suggesting that the reduced prodigiosin production bona fide stemmed from alterations in the related metabolism of *S. marcescens*. Of note, the spots of the LS group were below, but closer to, the linear standard curve, indicating a synergistic effect of larvae and *L. plantarum* on altering *S. marcescens* metabolism.

**Figure 3:**
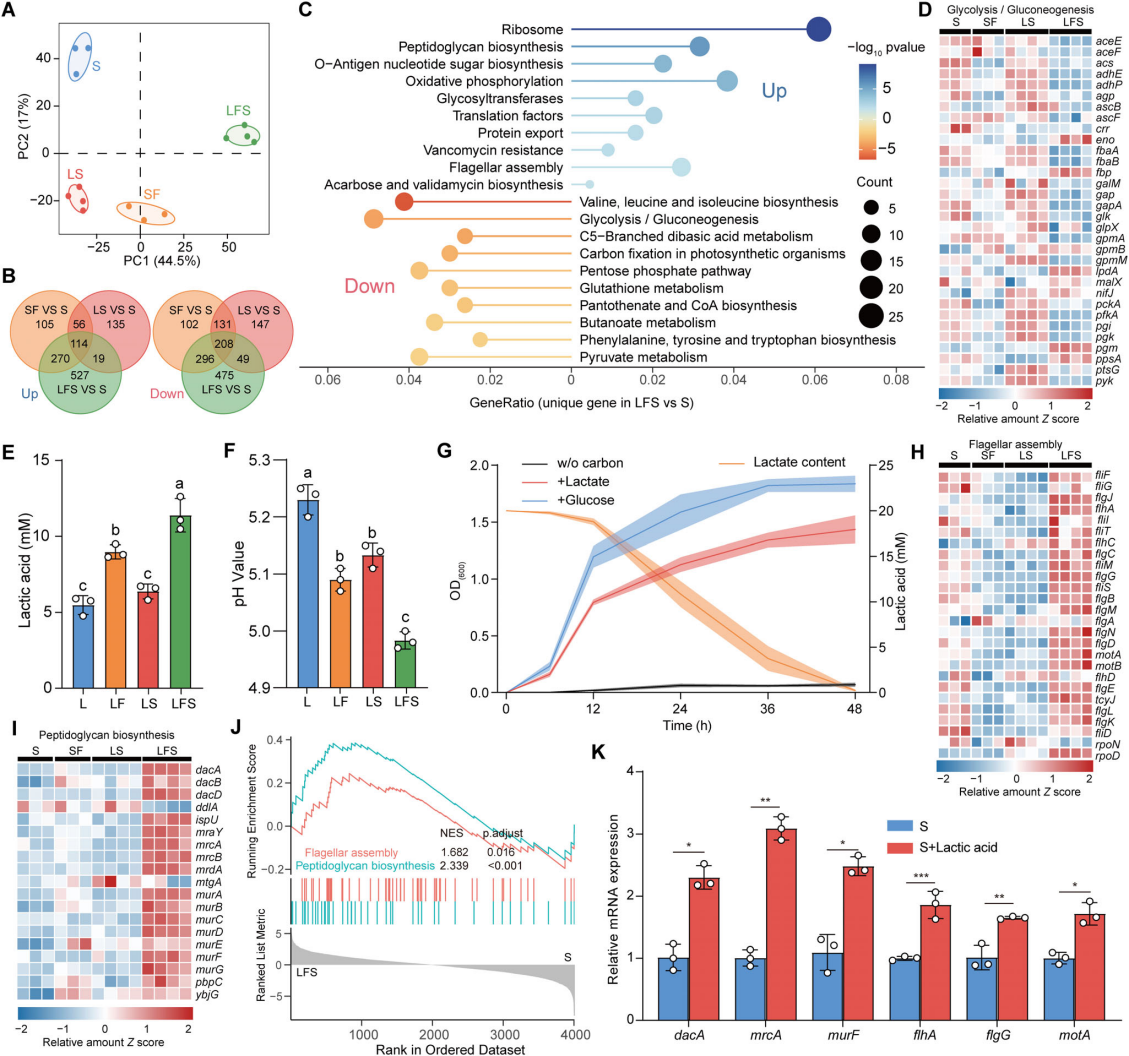
*Drosophila* larvae and *L. plantarum* enforced transcriptional profiling of *S. marcescens*. A) PCA of *S. marcescens* gene expression at 24 h timepoint after inoculation in S, SF (*S. marcescens* co‐cultured with the larvae), LS, and LFS groups. (*n*  =   3 – 4). B) Venn diagram showing the overlap of differentially expressed genes between groups (*n*  =   3–4). C) KEGG enrichment analysis was conducted on genes significantly upregulated (log2fold > 1; *p* < 0.05) and downregulated (log2fold < ‐1; *p* < 0.05) in *S. marcescens* within the LFS versus S, excluding the overlap of SF versus S and LS versus S. D) Heatmap of the expression profiles of glycolysis/gluconeogenesis genes in the SF, LS, and LFS groups versus S group. *Z* scores of the relative gene expression levels are displayed in the heatmaps (*n* = 3–4 independent experiments), with red representing higher and blue representing lower abundance. E) Lactate levels and F) pH value of *L. plantarum* after 24 h in L, LS, LF, and LFS (*n*  =   3). The data represent the means ± SD. The different letters above the columns in E and F denote statistically significant differences between groups (*p*  <  0.05). One‐way ANOVA followed by Tukey's test for multiple comparisons. G) The growth curve of *S. marcescens* (left y‐axis) and the concentration curve of lactate (right y‐axis) in the time course. *S. marcescens* was cultured in non‐carbon CDM alone, or supplemented with either glucose or L‐lactate. The OD values for bacterial growth and the lactate concentration were assessed separately over time. The solid line and shaded area of the curves represent the mean ± SD. H,I) Heatmap of the expression profiles of H) flagellar assembly and I) peptidoglycan biosynthesis genes in the SF, LS, and LFS groups versus S group. *Z* scores of the relative gene expression levels are displayed in the heatmaps (*n* = 3–4 independent experiments), with red representing higher and blue representing lower abundance. J) Gene set enrichment analysis (GSEA) analysis of the KEGG pathways of *S. marcescens* in the LFS group compared to the S group. K) qRT‐PCR was utilized to analyze the expression levels of resistance and virulence‐associated genes of *S. marcescens* in a fly diet with or without 20 mm lactate (*n* = 3). The data represent the means ± SD, and the significance analysis was performed by unpaired two‐sided Student's *t*‐test. *p <  0.05;**p  <  0.01; ***p  <  0.001.

To identify the set of genes specifically expressed during symbiosis, bulk RNA‐seq analysis of *S. marcescens* was performed. PCA and hierarchical clustering analysis (HCA) revealed that four conditions were strikingly different (**Figure** **3**A; Figure S3A, Supporting Information), while the replicates were similar within each condition. The global gene expression pattern of *S. marcescens* in the LFS group was most significantly different from the other three groups (Figure S3A, Supporting Information). Based on unique DEGs for the larvae and *L. plantarum* alliance (Figure 3B), pathway enrichment analysis showed that the expression pattern of *S. marcescens* was characterized by 10 most significantly upregulated and downregulated pathways (Figure 3C). Among these enriched pathways, attentions were paid to the downregulated glycolysis pathway that reverted to *L. plantarum* metabolism. To support it, the majority of genes in this pathway were downregulated (Figure 3D). Given that coexisting species are prone to circumvent the competition for ephemeral sugars, we predicted that *S. marcescens* could shift glucose to fermentable lactate produced by *L. plantarum*. To validate this prediction, we measured lactate in the medium. Unanticipatedly, the level of lactate was even higher in the LFS group than in the LF group (Figure 3E), accompanied by the lowest pH value (Figure 3F). This could be explained by the possibility that *S. marcescens* was unable to use lactate as the energy supply substrate. Alternatively, lactate production by *L. plantarum* overwhelmed lactate consumption by *S. marcescens*, resulting in a net increase in lactate. To address this issue, we investigated whether lactate was cross‐fed to *S. marcescens* in the liquid culture with lactate as the sole carbon source. Our data showed that lactate was almost depleted for the growth of *S. marcescens* (Figure 3G), supporting the concept of cross‐feeding between *L. plantarum* and *S. marcescens*. To rule out other metabolites, we performed a small‐scale screening. We found that pyruvic acid and glucose, commonly used as carbon and energy sources, robustly facilitated *S. marcescens* growth. Interestingly, lactate also substantially promoted it, although to a lesser extent than pyruvic acid and glucose (Figure S3B, Supporting Information). However, acetic acid, propionic acid, and butyric acid weakly supported this growth, while ethyl alcohol and acetaldehyde had no effect. Since pyruvic acid and glucose are frequently depleted in the niche, these results suggest that lactate could act as the sole signaling molecule. Notably, 20 mm of lactate reduced the medium pH to 5.2, but had only a minor inhibitory effect on *S. marcescens* load and pigment production (Figure S3C–F, Supporting Information). Lactate can act as the main fuel for the tricarboxylic acid (TCA) cycle and represents a central feature of metabolic remodeling.^[^ ^41^ ^]^ Intriguingly, the TCA cycle was not enriched in KEGG analysis, but the expression of several related genes was indeed upregulated (Figure S3G, Supporting Information). It could probably be explained by the average value defect of bulk RNA‐seq, which urgently requires a new technique to explore the gene expression heterogeneity hidden within populations.

Because the TCA cycle is a master regulator for virulence factor expression,^[^ ^42^ ^]^ changes in carbon source could potentially contribute to the heightened resistance of pathogens. As expected, lipopolysaccharide synthesis, peptidoglycan synthesis, and cationic antimicrobial peptide resistance were upregulated in the LFS group (Figure 3C). More importantly, gene heat maps showed that expression levels of genes in these three pathways were markedly higher in the LFS group than in the other two groups (Figure 3H,I). Gene set enrichment analysis (GSEA) showed apparent upregulation of these two pathways in the LFS group (Figure 3J). These findings provided a cue that *S. marcescens* could conduct its gene expression reprogramming to withstand the combined effects of host control and microbiota‐mediated colonization resistance. We confirmed the virulence of *S. marcescens* by measuring antimicrobial peptide expression in larvae using qPCR. Our results showed that *S. marcescens* alone indeed induced higher antimicrobial peptide gene expression in larvae than in either GF or *L. plantarum*‐associated larvae (Figure S4A, Supporting Information), consistent with a potential pathogen in *Drosophila*. Intriguingly, the expression levels of these genes in the LFS group were lower than those in the SF group, which contradicted the bulk RNA‐seq results. This might be explained by the possibility that the low proportion of *S. marcescens* in the total population didn't reach the threshold to trigger host immunity. Alternatively, subpopulations of *S. marcescens* exhibiting high virulence could artificially elevate the overall virulence of the total population, highlighting the crucial role of bacterial single‐cell transcriptomics in identifying subpopulations with high‐virulence gene expression. We further queried whether *S. marcescens* could overcome this colonization resistance by sensing lactate or other metabolites derived from *L. plantarum*. Indeed, lactate was more effective than any other metabolite in triggering the expression of virulence factors in *S. marcescens* (Figure 3K; Figure S4B, Supporting Information). Collectively, these findings suggest that lactate recapitulated *S. marcescens* response to compete with rivals of larvae and *L. plantarum*.

### 2.4 The Enforced Alterations in the Heterogeneity of Carbon Metabolism in *S. marcescens*

**Figure 4:**
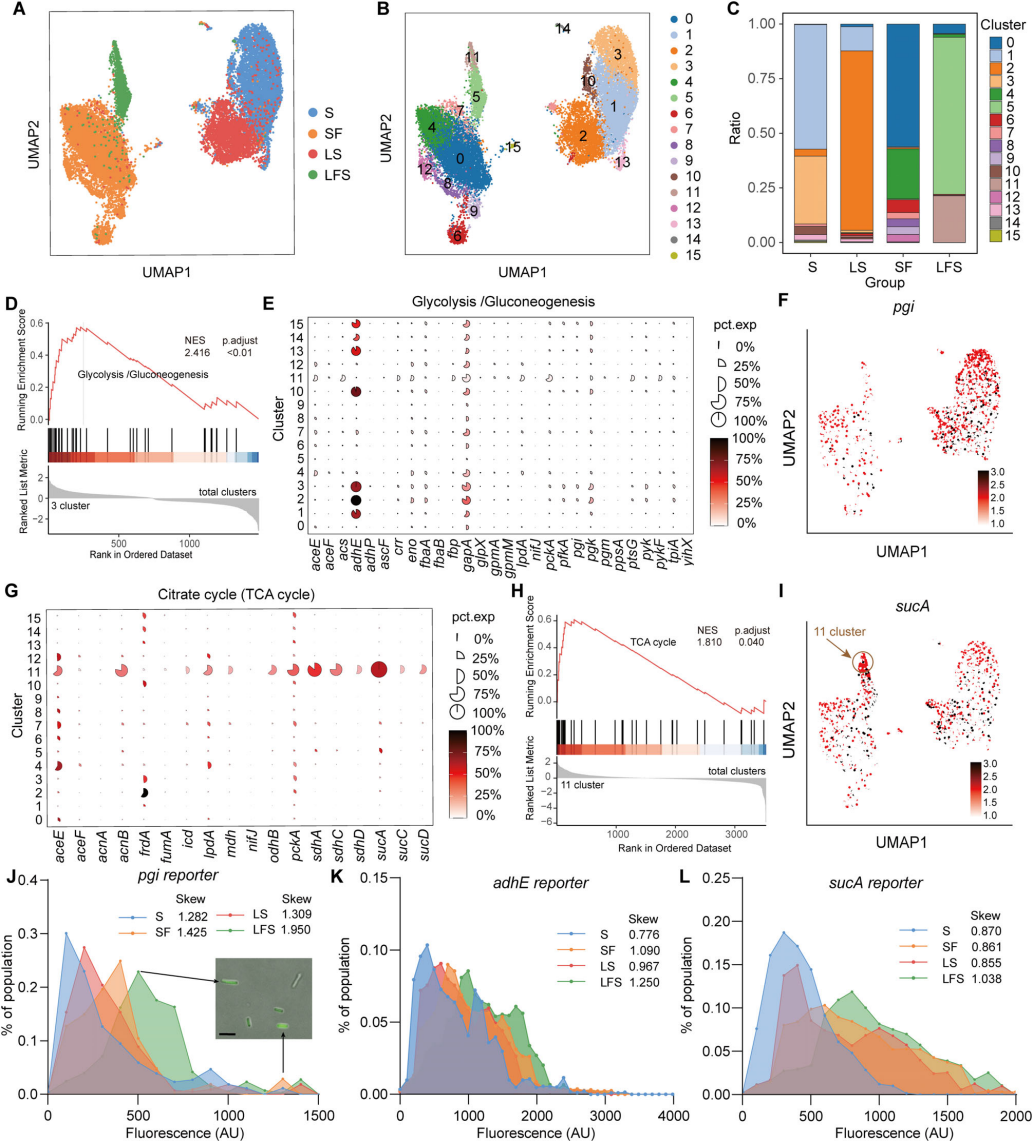
Larvae and *L. plantarum* induced phenotypic heterogeneity of *S. marcescens*. A,B) UMAP projection of all *S. marcescens* collected in S, SF, LFS, and LS groups, based on their gene expression colored by each group A). UMAP 2D representation of the 15 cell subclusters from all *S. marcescens* collected in S, SF, LFS, and LS groups B). C) The proportion of cell lineages of *S. marcescens* in S, LS, SF, and LFS groups. The colors correspond to the different cluster types. D) GSEA enrichment plots for the glycolysis/gluconeogenesis pathways upregulated in Cluster 3 compared to all clusters. E) Mean expression levels and proportions of glycolysis/gluconeogenesis genes in different subclusters. The shape of each dot indicates the proportion of cells in the cluster, while the color indicates the average activity normalized from 0% to 100% across all clusters. F) The expression of *pgi* genes was highlighted on the UMAP. G) Expression levels and proportions of genes in TCA cycle genes in different subclusters. H) GSEA enrichment plots for the tricarboxylic acid (TCA) cycle pathways upregulated in Cluster 11 compared to all clusters. I) The expression of *sucA* genes was highlighted on the UMAP. J–L) The line chart illustrates the heterogeneous expression of glycolysis‐associated genes J) *pgi*, K) *adhE* and L) a TCA cycle‐associated gene *sucA*. Representative images of each heterogeneous marker in the population as confirmed by fluorescent promoter‐reporter constructs (P‐*pgi*‐GFP, P‐*adhE*‐GFP, and P*‐sucA*‐GFP, respectively). Scale bars, 1 µm.

To understand the enforced alterations in the phenotypic heterogeneity of *S. marcescens* in response to the larvae and *L. plantarum* alliance, bacterial single‐cell RNA sequencing was performed on the existing platform.^[^ ^43^ ^]^ In total, we captured 19927 *S. marcescens* cells in four conditions with a median of over 200 genes per cell (Figure S5A,B, Supporting Information). Fascinatingly, each condition generated unique heterogeneity in global gene expression at the single‐cell resolution, visualized by graph‐based clustering of gene expression profiles (**Figure** **4**A). Unsupervised clustering analysis further unveiled 16 distinct clusters in the total cells (Figure 4B), and most clusters exclusively corresponded to one of the four conditions (Figure 4C). To uncover the heterogeneity of gene expression patterns, we performed an analysis of DEGs in 16 clusters (Figure S5C, Supporting Information). A pronounced enrichment of glycolysis in Cluster 3 (corresponding to the S group) caught our attention (Figure 4D), due to its consistency with the bulk RNA‐seq result (Figure 3D). To further understand alterations in the heterogeneity, we ranked marker genes related to glycolysis in each cluster. An increase in the proportion and expression levels of corresponding genes was observed in larva‐free subpopulations (Cluster 1, 2, 3, 10) compared to larva‐living ones (Cluster 0, 4, 5, 7, 11; Figure 4E). For example, the expression of *adhE*, *gapA*, and *pgi* genes involved in glycolysis was apparently higher in larva‐free Cluster 1, 2, 3, 10 than in larva‐living Cluster 0, 4 (Figure 4F; Figure S5D,E, Supporting Information). Moreover, the suppression of these genes in larva‐living clusters was further exacerbated by *L. plantarum* transplantation in Clusters 5 and 11. By contrast, the proportion and expression of genes involved in the TCA cycle were unevenly enhanced in larva‐living subpopulations, with a bias towards Cluster 4, 7, 11 (Figure 4G), which probably accounted for the failure in TCA cycle enrichment in the bulk RNA‐seq. Moreover, *L. plantarum* transplantation dramatically upregulated the expression of these genes in Cluster 11, and GSEA analysis showed that TCA cycle was highly enriched in Cluster 11 (Figure 4H). In contrast, the expression of TCA cycle‐related genes *sucA*, *acnB* and *sdhA* was higher in Cluster 11 (22.2%, 296/1352) than in Cluster 5 (Figure 4I; Figure S5F,G, Supporting Information), implicating that Cluster 11 might have developed from Cluster 5. To verify the previous results, key identified genes *pgi*, *adhE*, and *sucA* were selected, and constructed with a green fluorescent protein (GFP) reporter under the control of promoters, allowing us to examine their heterogeneous expression. Consistently, the average value of fluorescence intensity of *pgi* and *adhE* involved in glycolysis was higher in the larva‐free group than in the LFS group (Figure 4J,K). However, both *pgi* and *adhE* almost displayed unimodal distributions with a relatively lower skew in the S group, but dimodal distributions with a higher skew in LSF group, suggesting that larvae and *L. plantarum* contributed to more heterogeneity of glycolysis. In contrast, the average value of fluorescence intensity involved in the TCA cycle was lower in the larva‐free group than in the LFS group (Figure 4L). Similarly, the expression of *sucA* was more heterogeneous in the LSF group than in the S group. Overall, these findings suggest that the larvae and *L. plantarum* alliance could promote the division of the *S. marcescens* population into distinct metabolic subpopulations.

### 2.5 Changes of Heterogeneity in Virulence Gene Expression of *S. marcescens*

**Figure 5:**
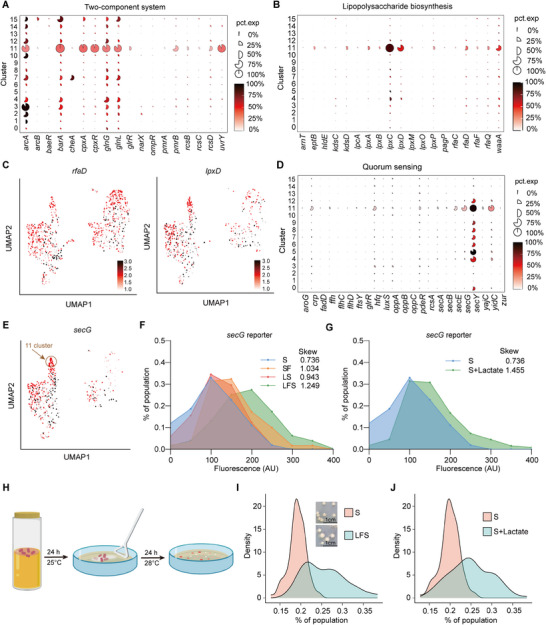
Larvae and *L. plantarum* induced heterogeneity of *S. marcescens*. A,B) Mean expression levels and proportions of genes in A) two‐component system and B) lipopolysaccharide biosynthesis pathways in different subclusters. C) The expression of *rfaD* and *lpxD* genes was highlighted on the UMAP. D) Mean expression levels and proportions of genes in quorum sensing genes in different subclusters. E) The expression of *secG* genes was highlighted on the UMAP. F) The line chart illustrates the heterogeneous expression of the virulence‐associated gene *secG* (P‐*secG*‐GFP). G) The line chart illustrates an increase in the expression heterogeneity of *secG* triggered by 20 mm lactate. H) Diagram of the heterogeneous resistance assay of colony size. Bacteria from the S and SLF groups were collected after 24 h of growth and spread on LB plates containing 40 mm tetracycline. Colony diameters were measured after an additional 24 h of incubation. I) The distribution of colony diameter sizes of *S. marcescens* in the S and LFS groups (*n* = 3). J) The distribution of colony diameter sizes of *S. marcescens* with or without 20 mm lactate (*n* = 3).

In response to selective pressure, pathogens can develop diverse strategies to exploit breakdowns in host control and colonization resistance.^[^ ^44^, ^45^ ^]^ To shed light on it, we evaluated whether the expression of two‐component systems changed in *S. marcescens* challenged with the host and *L. plantarum* rivals. Attractively, the proportion and expression levels of the two‐component system‐related genes, including *cpxA*, *pmrB*, and *uvrY*, were substantially increased in Cluster 11 compared to Cluster 5 (**Figure** **5**A). Mounting evidence has revealed that bacteria increase cell wall thickness, motility, and toxin secretion in response to unfavorable events.^[^ ^46^, ^47^ ^]^ Indeed, the proportion and expression levels of lipopolysaccharide synthesis and peptidoglycan synthesis‐related genes, including *rfaD*, *lpxD*, *mrcA*, and *dacA*, were substantially increased in Cluster 11 cells (Figure 5B,C; Figure S6A, Supporting Information). Furthermore, Cluster 11 remarkably exhibited upregulation of genes associated with movement, including those encoding bacterial motility protein (*cheA*, *flgK*, *pilW*) and flagellar assembly (*flgK*, *flhC*, *flhD*, *rpoD*, *rpoN*; Figure S6B,C, Supporting Information). Interestingly, we also identified a rare population of Cluster 7 (belonging to SF) that featured upregulation of the mobility‐related genes. Meanwhile, the expression levels of genes related to bacterial quorum sensing and secretion pathways, such as *secD*, *secE*, *secG*, and *yidC*, substantially increased in Cluster 11 cells (Figure 5D,E; Figure S6D, Supporting Information). To confirm these results, the heterogeneous expression of *secG*, a virulence‐associated gene, was evaluated as described above. Indeed, the average value of fluorescence intensity of *secG* was higher in the LFS group than in others (Figure 5F). Analogously, *secG* exhibited a distribution with a higher skew in the LSF group, suggesting that larvae and *L. plantarum* contributed to increased heterogeneity in virulence gene expression of *S. marcescens*. Given that lactate triggered the expression of most virulence genes in *S. marcescens* (Figure 3), we were further prompted to investigate whether lactate could induce increased heterogeneity in *secG* expression upon lactate treatment. Indeed, a distribution with a higher skew was observed in the lactate‐treated group compared to the untreated one (Figure 5G). These findings suggest that S. *marcescens* precisely regulates the expression of virulence genes by sensing lactate.

Colony size variation is prevalent in bacterial growth, making it an effective proxy for assessing resistance heterogeneity.^[^ ^48^ ^]^ We devised a method to assess heterogeneity within the bacterial population by examining the colony sizes of *S. marcescens* on tetracycline‐containing plates as illustrated (Figure 5H). The data showed that the proportion of large colony sizes increased after *S. marcescens* had been inflicted with a couple of larvae and *L. plantarum* (Figure 5I). This suggests that *S. marcescens* in the LFS group exhibited heterogeneous resistance, with some cells growing more rapidly on the antibiotic plates. Moreover, we observed an increased proportion of large colony sizes after *S. marcescens* had been exposed to lactate (Figure 5J). Taken together, these findings demonstrate that *S. marcescens* can alter the heterogeneity of virulence gene expression in response to competitive pressures, thereby acquiring a fitness advantage in a competing niche.

### 2.6 *L. plantarum* Alters the Heterogeneity of Glycolysis in Adaption to the Alliance

**Figure 6:**
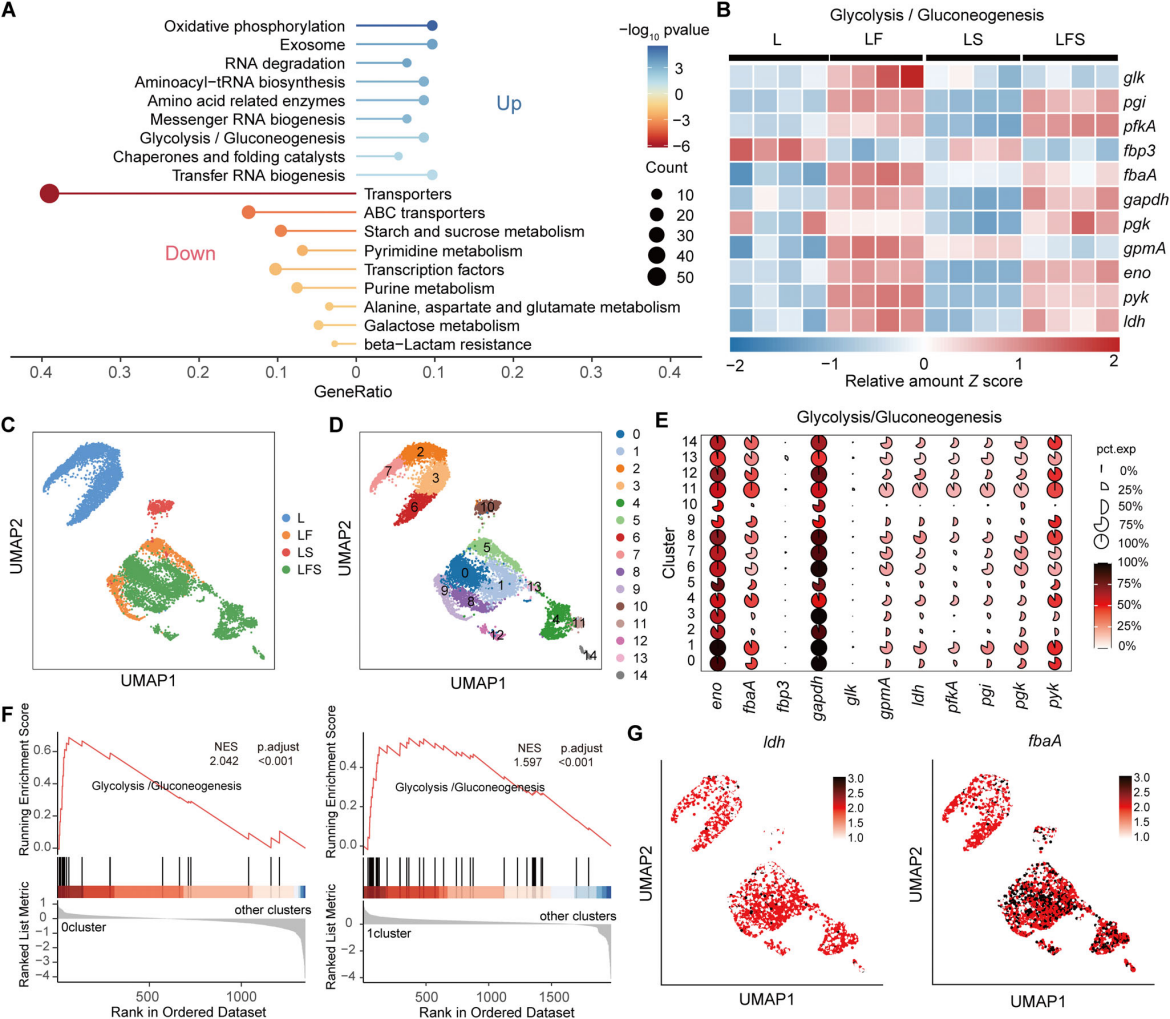
*Drosophila* larvae stimulated lactate synthesis of *L. plantarum*. A) KEGG enrichment analysis of the significantly upregulated (log_2_fold > 1; *p* < 0.05) and downregulated (log_2_fold < ‐1; *p* < 0.05) genes in *L. plantarum* in the LFS group compared to the L group. B) Heatmap of the expression profiles of glycolysis/gluconeogenesis genes of *L. plantarum* in L, LF, LS, and LFS groups. *Z* scores of the relative gene expression levels are displayed in the heatmaps (*n* = 3–4 independent experiments), with red representing higher and blue representing lower abundance. C,D) UMAP combined *L. plantarum* cell data colored by C) treatment and D) 15 cell clusters. E) Mean expression levels and proportions of glycolysis/gluconeogenesis genes in subclusters. The shape of each dot indicates the proportion of cells in the cluster, while the color indicates the average activity normalized from 0% to 100% across all clusters. F) GSEA enrichment plots for glycolysis/gluconeogenesis pathways upregulated in Cluster 0 and 1. G) The expression of two representative genes of glycolysis/gluconeogenesis was highlighted on the UMAP.

To better understand how *L. plantarum* joined the alliance to resist *S. marcescens*, we first analyzed the transcriptomic profiling of *L. plantarum* in bulk. Both PCA and HCA revealed the unique gene expression pattern in the LFS group (Figure 1B; Figure S7A, Supporting Information). The glycolysis pathway was enriched in the LFS group compared to either larvae‐free group (**Figure** **6**A; Figure S7B, Supporting Information). Moreover, expression levels of glycolysis‐related genes were significantly upregulated compared to the larvae‐free groups (Figure 6B), which was verified by qPCR analysis (Figure S7C, Supporting Information). Intriguingly, expression levels of glycolysis‐related genes in the LFS group were comparable to those in the LF group. However, host‐triggered lactate production of *L. plantarum* was further raised in the presence of *S. marcescens* (Figure 3E). This contract could be partially explained by the higher load of *L. plantarum* or by glycolytic heterogeneity within the population.

To explore changes in glycolysis heterogeneity in *L. plantarum*, we analyzed the transcriptome of single bacterial cells. This endeavor resulted in a comprehensive dataset comprising 8290 individual *L. plantarum* cells (Figure S7D,E, Supporting Information). The results show that larvae, *S. marcescens* or both induced differential transcription patterns of *L. plantarum* at the single‐cell resolution (Figure 6C,D). Consistent with the bulk results of upregulated glycolysis, we ranked marker genes associated with glycolysis in each cluster (Figure 6E), and observed the heterogeneity with a relative increase in the proportion and expression levels of corresponding genes within larvae‐free populations (Cluster 2, 3, 10) and larvae‐living populations (Cluster 0, 1, 5). Moreover, levels of upregulated expression were higher in Cluster 0 and 1 than in Cluster 5, which appeared to contradict the bulk result of comparable glycolysis. To ease the tension, the heterogeneity of lactate production in the LFS group was further analyzed. The proportion and expression levels of corresponding genes were lower in Cluster 4 than in Cluster 0, and 1, which possibly undermined the overall level of glycolysis. Indeed, the GSEA analysis showed significant enrichment in glycolysis was merely observed in Cluster 0, 1 belonging to LFS (Figure 6F). The heterogeneity in the expression of glycolysis‐related genes *ldh* and *fbaA* was higher in Cluster 0 and 1 (belonging to the LFS group) than in Cluster 5 and 9 (belonging to the LF group, Figure 6G). Notably, the ubiquitous expression of glycolysis genes was observed across all clusters, indicating that glycolysis is a fundamental metabolic pathway essential for *S. marcescens* survival and energy production. Altogether, these results suggest that the *L. plantarum* population engage in the alliance to resist *S. marcescens* by changing the heterogeneity of glycolysis.

## 3 Discussion

**Figure 7:**
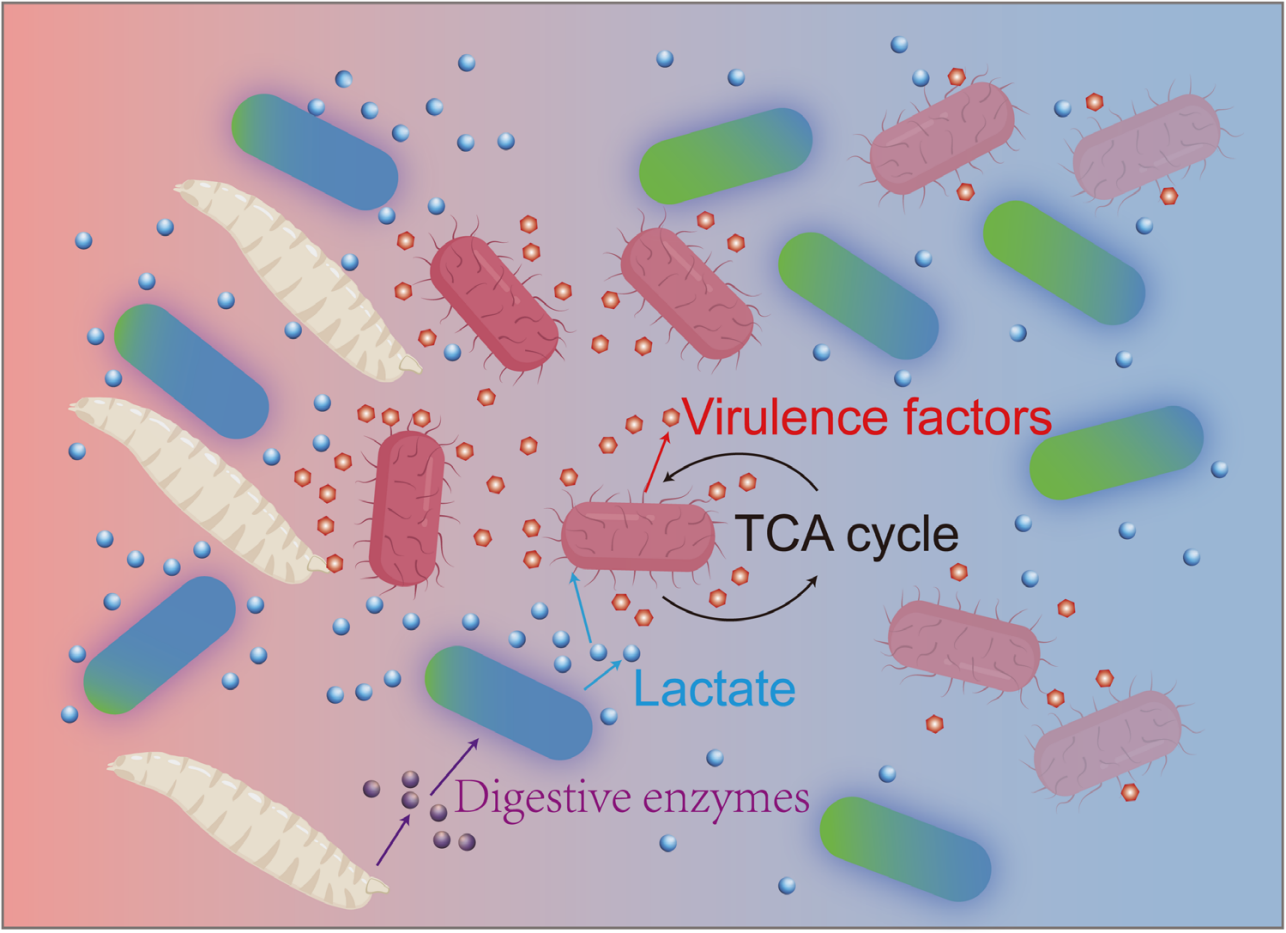
Conceptual model of interactions in the tripartite *Drosophila*‐*L. plantarum*‐*S. marcescens* model. *Drosophila* larvae stimulate lactate production by *L. plantarum* through excreting digestive enzymes. *S. marcescens* cells sense lactate cues in their environment and adapt metabolically in a heterogeneous fashion.

Hosts have evolved a myriad of mechanisms to monitor their symbionts in order to maximize benefits from them. Understanding the ecological dynamics of multiple species is integral for precise bacterial manipulation. In this study, we showed that larvae specifically promoted the growth of *L. plantarum* in the presence of a pathogen, but thwarted the propagation of *S. marcescens* by cooperation with *L. plantarum*. The results suggest that the host modifies the environment, like social digestion, to facilitate the growth of commensals in natural communities, and forms an alliance with commensals to combat pathogen invasion. As initially dominant in the niche, *L. plantarum* rapidly consumed the preferred carbon source, which rendered it extremely competitive by restricting carbon source availability to surrounding bacteria (**Figure** **7**). Aside from participation in the alliance, *L. plantarum* engaged in a niche with a vast metabolic repertoire and exerted antimicrobial effects on pathogens (Figure 3E). As a simple fermentation end product, lactate can be widely generated by many bacterial species,^[^ ^49^ ^]^ and is a central feature of metabolic remodeling. However, the single‐cell RNA‐seq analysis showed heterogeneity of lactate generation in the *L. plantarum* subpopulation (Figure 6E–G). Combined with the long‐term relationship of *L. plantarum* and *Drosophila*,^[^ ^50^ ^]^ these results demonstrated that commensals participated in the fine‐tuning of the global transcriptional profile in the adaptation in the alliance with larvae to resist pathogens (Figure 7). Our model system provides a reductionist approach to disentangle the inherent complexity of interplay inside bacterial communities. However, considering the diverse composition of the natural microbiota, conducting investigations of wild‐caught larvae is imperative to improve our understanding of the ecology of host‐symbiont interactions in future studies.

*S. marcescens* is an opportunistic pathogen causing morbidity and mortality in the developing world, mainly due to its virulence factors.^[^ ^51^ ^]^ Pathogens have developed sophisticated strategies to overcome host control and colonization resistance.^[^ ^5^, ^52^ ^]^ Although suppressed by the host and commensal alliance, *S. marcescens* grew faster than *L. plantarum* in the medium, reaching approximately the half‐load of the total bacteria 24 h following inoculation (Figure 2A,B). Pathogens invading this ecosystem are likely to have evolved unique metabolic adaptations to circumvent nutritional competition with commensal microbes.^[^ ^53^, ^54^ ^]^ One of the metabolites lactate regulates the growth and triggers the expression of virulence factors in various pathogenic bacteria.^[^ ^55^, ^56^ ^]^ Hence, a dynamic regulation of virulence factor expression may be a key invasion strategy for *S. marcescens* in response to environmental cues by cross‐feeding.^[^ ^57^ ^]^ We indeed found that *S. marcescens* robustly exerted the transcriptional reprogramming to express many virulence factors in response to larvae and *L. plantarum* (Figure 3C). Moreover, pathogens expressed virulence factors in multiple fashions or phenotypic heterogeneity (Figure 5). Nevertheless, bulk RNA‐seq analysis evidently masked phenotypic heterogeneity of microbial communities, such as carbon metabolism adaptation in Cluster 11 cells and virulence and pathogenicity‐related gene expression in that cluster. By applying this technique to interrogate populations on the single‐cell level, we observed that *S. marcescens* single cells were distinct from each other concerning gene expression when they encountered larvae and *L. plantarum* (Figure 4). The findings supported the proposal that a subpopulation of pathogens was required to express virulence factors for competition with resident microbes.^[^ ^22^, ^58^ ^]^ Seemingly identical bacterial cells in a population manifested phenotypic heterogeneity in terms of antibiotic susceptibility, highlighting novel microbiome‐targeted therapies designed to cope with the infection of life‐threatening pathogens.^[^ ^59^, ^60^ ^]^ The application of a single‐cell resolution approach enables a deeper understanding of how lactate modulates heterogeneous microbial behavior at the individual cell level, opening the door to targeted therapeutic strategies against persister cells in recalcitrant bacterial infections. Given the existence of certain *S. marcescens* subpopulations exhibiting resistance to colonization resistance, we propose that identifying alternative inhibitors specifically targeting this subpopulation could provide an effective strategy to overcome such resistance. For instance, our previous study demonstrated that antimicrobial peptides can generally reduce the resistance of *S. marcescens*.^[^ ^34^ ^]^ It would be particularly interesting to investigate whether combining lactate with AMPs or inhibitors targeting newly identified genes could enhance the elimination of pathogens from the gut.

In summary, we developed an integrative and synthetic model that revealed tripartite interactions between commensals, pathogens, and the host. These findings expand our understanding of the internal regulatory networks at the single‐cell resolution, opening up new avenues for the development of innovative approaches to prevent pathogens in the future.

## 4 Experimental Section

### Drosophila *Stocks and Dietary Treatments*

*Stocks and Dietary Treatments*: All experiments were performed with wild‐type Canton‐S (wtcs) flies and reared under controlled conditions at 25 °C, 60% humidity, and 12  h light/dark cycle. Flies were kept in vials containing standard cornmeal agar medium. The standard food recipe was as follows: 64 g dextrose, 9 g agar, 25 g yeast, 65 g cornmeal, 32 g sucrose, and 1 L purified H_2_O were mixed and boiled for 10 min with constant agitation, and 0.51 g Sodium benzoate (Sigma Aldrich) dissolved in 1 mL 95% ethanol and 2 mL propionic acid (99%, Mallinckrodt Baker) was added. Germ‐free fly stocks were maintained on sterilized high yeast‐based medium (high YBM) with the following proportions: 64 g dextrose, 12 g agar, 50 g yeast, 65 g cornmeal, and 32 g sucrose.

### Generation of GF Flies

GF flies were generated as previously described.^[^ ^3^ ^]^ Briefly, embryos were collected from agar media and washed successively with ddH_2_O, Walch sanitizer (1:30, Procter & Gamble Co.), 2.5% sodium hypochlorite (Sigma Aldrich), 75% ethanol, and sterile PBS containing 0.01% Triton X‐100 before being transferred into axenic food vials. Confirmation of GF larvae was achieved by performing 16S rDNA PCR on homogenates of adult flies or by plating the homogenates on nutrient agar plates (beef extract powder 3 g L^−1^; peptone 10 g L^−1^; NaCl 5 g L^−1^; agar 15 g L^−1^).

### Bacterial Strains and Growth Curves

All materials to manipulate bacteria were sterilized before usage. Strains of *Serratia marcescens* with the Genbank accession number CP053378 and *Lactiplantibacillus plantarum* with the Genbank accession number KY038178 were used. Unless otherwise stated, *S. marcescens* and *L. plantarum* were cultured overnight at 30 °C in LB and MRS broth, respectively. For growth curves, bacteria were cultured overnight at 30 °C in CDM (chemically defined medium) supplemented with 20 mm of glucose, pyruvic acid, ethanol, acetaldehyde, acetic acid, propionic acid, butyric acid, or L‐lactate.^[^ ^61^ ^]^ The OD_600_ values of the cultures were determined accordingly.

### Microbial Growth Kinetics of Single, Mono‐ and Diassociations

Single or dual bacterial cultures (0.1 OD_600_ in total) were inoculated into fly food either without or with forty 4‐day‐old germ‐free larvae to generate mono‐ or di‐associated flies as previously described.^[^ ^34^ ^]^ To ensure the same total number of inoculated cells, the OD_600_ values of the cultures with a spectrophotometer (UV‐2450, Shimadzu, Japan) were measured and a total standardized inoculum equivalent to 0.1 OD_600_ (approximately 10^7^ CFU mL^−1^) through appropriate dilutions prior to inoculation was calibrated. For mono‐associations, fresh stationary phase bacterial cultures (0.1 OD_600_) were added directly to fly vials containing 5 mL food. Associations with two bacteria (0.1 OD_600_) were performed in a mixture at *L. plantarum*: *S. marcescens* ratios of 1:10^−4^, 1:10^−2^, 1:1, 10^2^:1, 10^4^:1. To accurately quantify bacterial loads in the medium, larvae were removed prior to assessment. Briefly, 5 mL of sterile PBS was added to each vial, and the entire food content was subjected to vigorous stirring before being transferred to 15 mL tubes. Bacterial loads were determined by plating 10‐fold serial dilutions of the homogenates on LB or MRS agar plates and incubated at 30 °C for *S. marcescens* and *L. plantarum*, respectively. Colony‐forming units (CFU) were counted after 24 h of incubation.

### pH Value, Lactate and Prodigiosin Production Assay

The pH value of the medium was measured at a depth of 50 mm using a pH meter (FE28, Mettler) with an accuracy of 0.01. The lactate concentration in the diet was determined using a lactate test kit (Nanjing Jiancheng Bio Inc.), following the manufacturer's instructions. Prodigiosin levels were measured according to previously reported methods.^[^ ^38^, ^40^ ^]^ All experiments were independently replicated three times.

### External Digestion Enzyme Treatment

For the collection of larval saliva, 4 d GF larvae were collected and rinsed in PBS three times. Forty larvae were bathed in 2 mL of PBS and kept overnight at 25 °C. After incubation, larval saliva was collected after removing the larvae. Larval saliva (1 mL), sucrase (Sigma Aldrich, 20 units mL^−1^), amylase (Sigma Aldrich, 20 units mL^−1^), or both enzymes (each at a final concentration of 20 units mL^−1^) was respectively added to 5 mL High YBM. Simultaneously, *L. plantarum* (0.1 OD_600_) was inoculated into the medium. The lactate content and bacterial load in the medium were measured after 24 hours as described above.

### Real Time‐PCR Analysis

*D. melanogaster* RNA was isolated from 10 larvae using TriZol reagent (Thermo Fisher Scientific). Bacterial cells were collected from fly food as previously described,^[^ ^34^ ^]^ and total RNA was extracted using the Bacterial RNA Extraction Kit (Vazyme, China). The RNA concentration was measured using a NanoDrop spectrophotometer (Thermo Fisher Scientific). Subsequently, 0.6 µg of total RNA was used for reverse transcription with the HiScript III All‐in‐One RT SuperMix Kit (Vazyme). The mixture was subjected to RT‐qPCR analysis using the ChamQ Universal SYBR qPCR Master Mix Kit (Vazyme) in a CFX96 Real‐Time System (BioRad, Hercules, CA, USA). Relative expression values were calculated using the following formula: △*C*t = *C*t (target gene) – *C*t (reference gene), and the relative expression was equal to 2^−△△^ *^C^* ^t^. The 16S rRNA gene served as an internal control. The primers used for RT‐qPCR analysis are listed in Tables S1 and S2 (Supporting Information). The experiments were independently replicated three times.

### Bacterial Bulk RNA Sequencing and Analysis

Bacterial cells were collected and total RNA extraction was prepared as previously described.^[^ ^34^ ^]^ Library construction was performed using VAHTS Universal V8 RNA‐seq Library Prep Kit for Illumina (Vazyme, China). The sequences were sequenced using Novaseq‐PE150 Novogene, China. The filtered clean reads were aligned to the reference sequence using Hisat2 (v. 2.1.0) to calculate the gene alignment rates. The clean reads were aligned to composite reference genomes of *S. marcescens* (GCF_013122155.1) and *L. plantarum* (GCF_032436005.1). Afterwards, read counts were used to quantify the expression levels of transcripts using FeatureCount (v. 2.0.1). The read counts for gene values in each species were used for analysis with DESeq2 (v. 1.30.0). The significant differentially expressed genes were determined in different groups using the DESeq software (DESeq2), with the standards of *P*‐value ≤ 0.05, and fold change. Functional analysis of genes was performed by transferring annotations from the eggNOG mapper. KEGG pathway enrichment was tested using the hypergeometric distribution. Gene Set Enrichment Analysis (GSEA) was conducted using ClusterProfiler (version 4.0.2) to identify enriched pathways based on KEGG terms. GSEA was performed with an FDR cutoff of 0.05 and a nominal *p* value of 0.05 or less.

### Bacterial Single‐Cell RNA‐Seq and Analysis

Bacterial single‐cell RNA sequencing was carried out as previously described.^[^ ^34^, ^43^ ^]^ In brief, bacterial cells were collected and fixed in 2 ml ice‐cold 4% formaldehyde (Sigma, 47608) with shaking overnight at 4 °C. Fixed cells were washed twice with 1x PBS supplemented with 0.1% Tween‐20 and 0.1 U mL^−1^ Murine RNase inhibitor (Thermo Fisher Scientific, Cat. N8080119). The supernatant was removed and cells were resuspended in 200 µL PBS‐TRI (1x PBS supplemented with 0.1% Tween‐20 and 0.2 U mL^−1^ Murine RNase inhibitor). Cell walls were digested with lysozyme (Thermo Fisher, Cat. 90082) at 37 °C for 15 min. Bacterial cells were subsequently washed and resuspended in PBS with RNase inhibitor. The cell suspensions were counted with a Moxi cell counter and diluted according to the manufacturer's instructions to obtain single cells. The bacterial single‐cell RNA‐Seq library was prepared according to the protocol of VITAPilote kit (M20 Genomics, R20114124). In situ reverse transcription of bacteria was performed with random primers and the resulting cDNA fragment was added with adaptor. The droplet barcoding for a single bacterium was performed on VITACruiser Single Cell Partitioning System (M20 Genomics, Hangzhou, China). Bacteria, DNA extension reaction mix, and hydrogel barcoded beads were encapsulated using the VITACruiser. The cDNAs were amplified by PCR, and purified with magnetic beads. All products were pooled to construct a standard sequencing library. Sequencing was done on a PE150 (Illumina), and raw reads were aligned to the genome of the species of interest using STAR (v. 2.7.10a) with default parameters. Read summarization was performed using the featureCounts (v. 2.0.1). The UMI count for each gene was determined using the UMI tools (v. 1.1.2). A matrix of gene counts was then generated for each cell (N‐by‐K matrix, with N cells and K genes). The quality of data was examined in Table S3 (Supporting Information). The single‐bacterium gene expression matrix of filtered data was then analyzed by R package Seurat 4 (v. 4.0.3). Genes were excluded that were not expressed in any cells in the dataset, and cells were filtered to retain only those with at least 100 genes and fewer than 1000 reads. The standard Seurat workflow prior to clustering was used including global normalization, feature selection, and scaling of gene expression. The top 2000 highly variable genes were used as input features for clustering analysis and downstream annotation. The Seurat packages FindNeighbors and FindClusters were used for clustering at a resolution of 0.5. Uniform manifold approximation and projection (UMAP) was utilized for the visualization of clustering. Differential gene expression (DEG) testing was performed using the function FindAllMarkers () under RNA assay mode in Seurat with a Wilcoxon rank‐sum test and p values were adjusted using Bonferroni correction. DEGs were filtered using a minimum log_2_ (fold change) of 0.5 and a maximum adjusted p‐value of 0.05. GSEA was also conducted by clusterProfiler (v. 4.0.2) based on KEGG terms. GSEA was performed with an FDR cutoff of 0.05 and nominal p value of 0.05 or less.

### Plasmid Construction and Fluorescence Intensity Assay

Recombinant expression plasmids containing the promoters of identified genes fused to *GFP* were constructed as previously described.^[^ ^62^ ^]^ Briefly, the promoters of pgi, adhE, sucA, and secG were amplified using the corresponding primers (Table S1, Supporting Information), along with the *GFP* gene from the pET28a:GFP plasmid. The promoter fragments were fused to GFP through overlap extension PCR and subsequently inserted into the linearized pBAM2 vector using the ClonExpressII One Step Cloning Kit (Vazyme Biotech Co.). The recombinant plasmid, pBAM2‐Promoter‐GFP, was transformed into *S. marcescens* electrocompetent cells using a MicroPulser Electroporator (Bio‐Rad), and positive clones were selected on LB agar plates containing 50 µg mL^−1^ gentamicin. *S. marcescens* carrying the reporter constructs were cultured overnight. Cells were collected, prepared, and visualized using a confocal microscope with a 100× oil‐immersion objective (Nikon AX/AX‐R). Fluorescence intensity was quantified using ImageJ software (v1.53, Bethesda, MD).

### Bacterial Colony Growth Heterogeneity Measurement

To assess bacterial colony growth heterogeneity, bacteria from different groups were incubated for 24 h. Then, 2 mL of ice‐cold 1×PBS was added to the vials to resuspend the bacterial cells for 5 min. The bacterial suspension was separated from residual food by centrifugation (900 rpm, 3 min) and washed twice with 1×PBS. The cells were collected by centrifugation (5000 rpm, 4 min) and washed again twice with 1×PBS. Subsequently, the cells were incubated in LB medium containing 40 mM tetracycline for 24 h. Finally, colony diameters were measured after an additional 24 h incubation.

### Statistical Analysis

Data were shown as mean ± standard deviation (SD) or box plot. Sample sizes (*n*) for each statistical analysis were depicted in the figure legends. To determine the significance of differences, whether the data were normally distributed using the D'Agostino–Pearson normality test was first assessed. If the data were normally distributed, data were analyzed using a Student's t‐test, one‐way ANOVA or two‐way ANOVA followed by Tukey multiple comparisons, as stated in the figure legends. If the data were not normally distributed, nonparametric tests were employed: two‐tailed Mann–Whitney U‐test for two groups or Kruskal‐Wallis test. For correlation analyses, linear regression was utilized. Significant differences were indicated using compact letter displays (CLD). First letters were assigned to groups based on the results of Tukey's test, with the group having the largest mean assigned the letter “a.” Groups that shared the same letter were not significantly different from each other (*p* > 0.05), while groups with different letters were significantly different (*p* < 0.05). All statistical analyses were performed using GraphPad Prism 8.0. Figures were produced using Adobe Illustrator CC 2020.

## Conflict of Interest

The authors declare no conflict of interest.

## Supporting information



Supporting Information

## Data Availability

The authors declare that all data supporting the findings of this study are available within this article. The bulk RNA‐seq data and single‐cell RNA‐seq data can be accessed at the GEO: GSE251783 (https://www.ncbi.nlm.nih.gov/geo/query/acc.cgi?acc=GSE251783).
