# Supplementary material for: Hosts and Commensal Bacteria Synergistically Antagonize Opportunistic Pathogens at the Single‐Cell Resolution
Source: Adv Sci (Weinh). 2025 May 23;12(30):e00582. doi: 10.1002/advs.202500582 (PMC12376580; doi:10.1002/advs.202500582)
Supplement: Supplementary file 1 — Supporting Information [file ADVS-12-e00582-s001.docx]

**Supporting information for**

**Hosts and Commensal Bacteria Synergistically Antagonize Opportunistic Pathogens at the Single-Cell Resolution**

Sheng Zhang, Ziguang Wang, Anqi Liu, Jinshu Li, Jingjing Zhuang, Xiaowen Ji, Paul I. Mulama, Maoye Li, Haiqun Cao, Eng-King Tan, Wei Liu*

**This PDF file includes:**

Figures S1 to 7

Tables S1 to 3


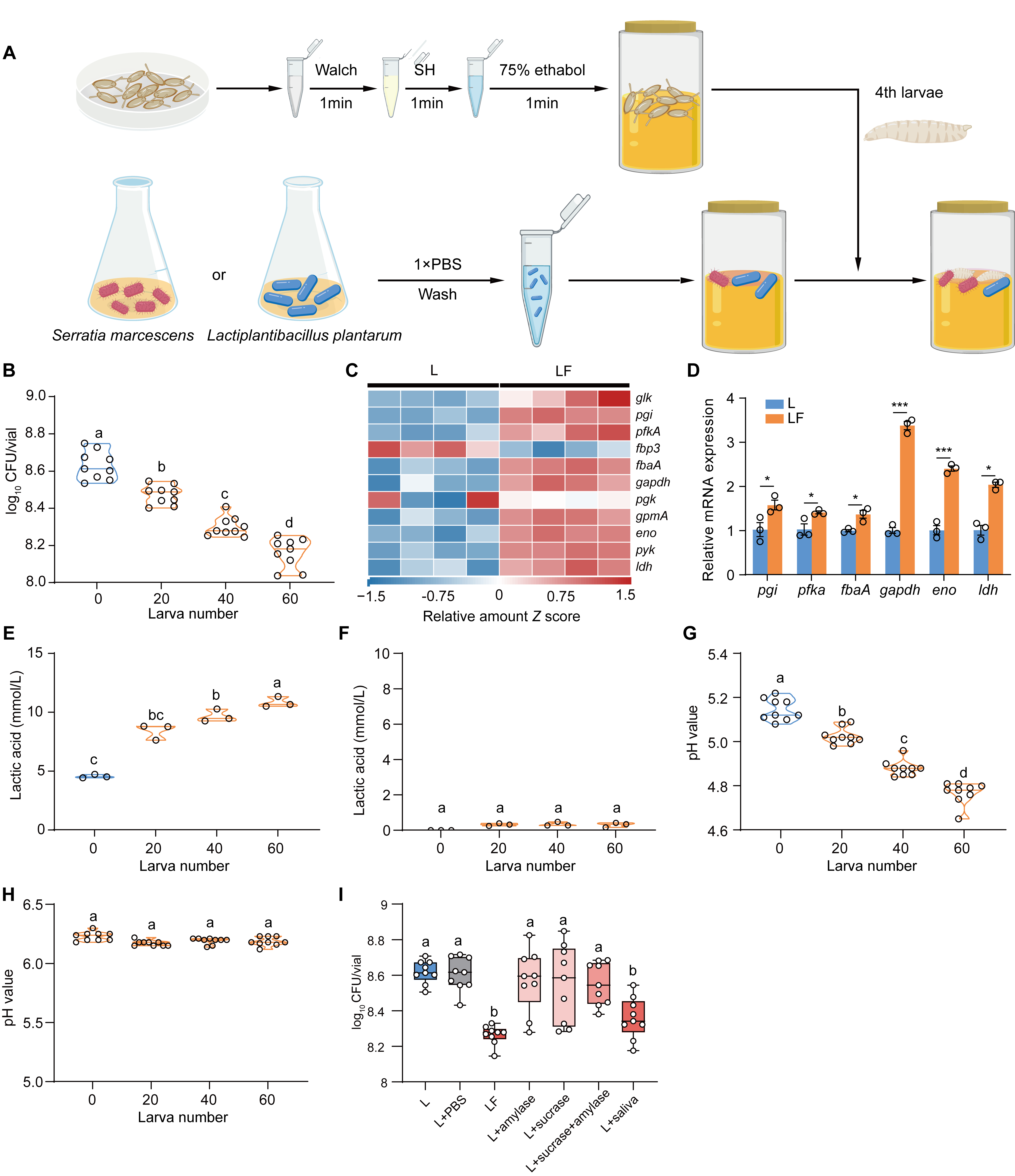


**Figure S1.** Host-stimulated lactate synthesis of *L. plantarum*. A) A diagram of a reductionist approach to investigate the causal role of *Drosophila* in regulating the physiology and behavior of bacteria. Top: Germ-free *Drosophila* larvae were generated by successive sterilization of fresh eggs with sanitizer Walch, sodium hypochloride (SH), ethanol, and PBS containing 0.01% TritonX-100T (PBST). Bottom: bacteria were cultured in a liquid medium and re-inoculated to fly cornmeal food after washing with PBS buffer. In the meantime, GF crawling larvae were transferred to the fly medium in the shared vials with bacteria. B) The bacterial load of *L. plantarum* in coculture with different numbers of crawling larvae (*n* = 3). C) Heatmap of the expression profiles of some glycolysis genes in the LF group compared to in the L group. *Z* scores of the relative gene expression levels are displayed in the heatmaps (*n* = 4 independent experiments), with red representing higher and blue representing lower abundance. D) qRT-PCR was utilized to analyze the relative expression levels of glycolysis-related genes normalized to the level of 16S rRNA transcripts (*n* = 3). The data represent the means ± SD, and the significance analysis was performed by unpaired two-sided Student’s *t*-test. **P*  <  0.05; ****P*  <  0.001. E-H) Examination of lactic acid levels (E-F) and pH values (G-H) in the medium with different numbers of crawling larvae co-cultured with *L. plantarum* or without *L. plantarum* (*n* = 9). I) The bacterial load of *L. plantarum* in supplemented with PBS, saliva, sucrase or amylase (*n* = 9). The different letters above the columns denote statistically significant differences (p < 0.05) between groups (one-way ANOVA followed by Tukey’s test for multiple comparisons) (B, E-I).


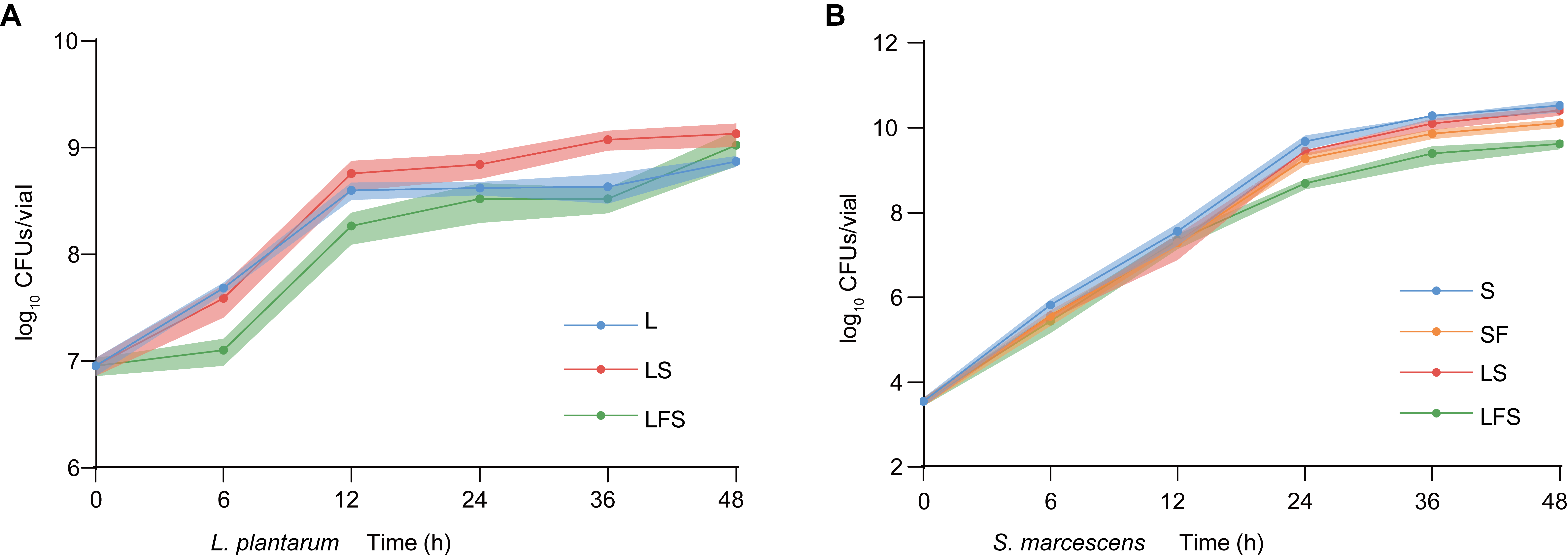


**Figure S2.** *Drosophila* larvae and *L. plantarum* independently enforced loading of *S. marcescens*. A) The bacterial load of *L. plantarum* over time in L, LS, LFS group. (*n*  =  9). The solid line and shaded area show mean ± SD, respectively. B) The bacterial load of *S. marcescens* over time in S, SF, LS, and LFS group (*n*  =  9). The solid line and shaded area show mean ± SD, respectively.

**
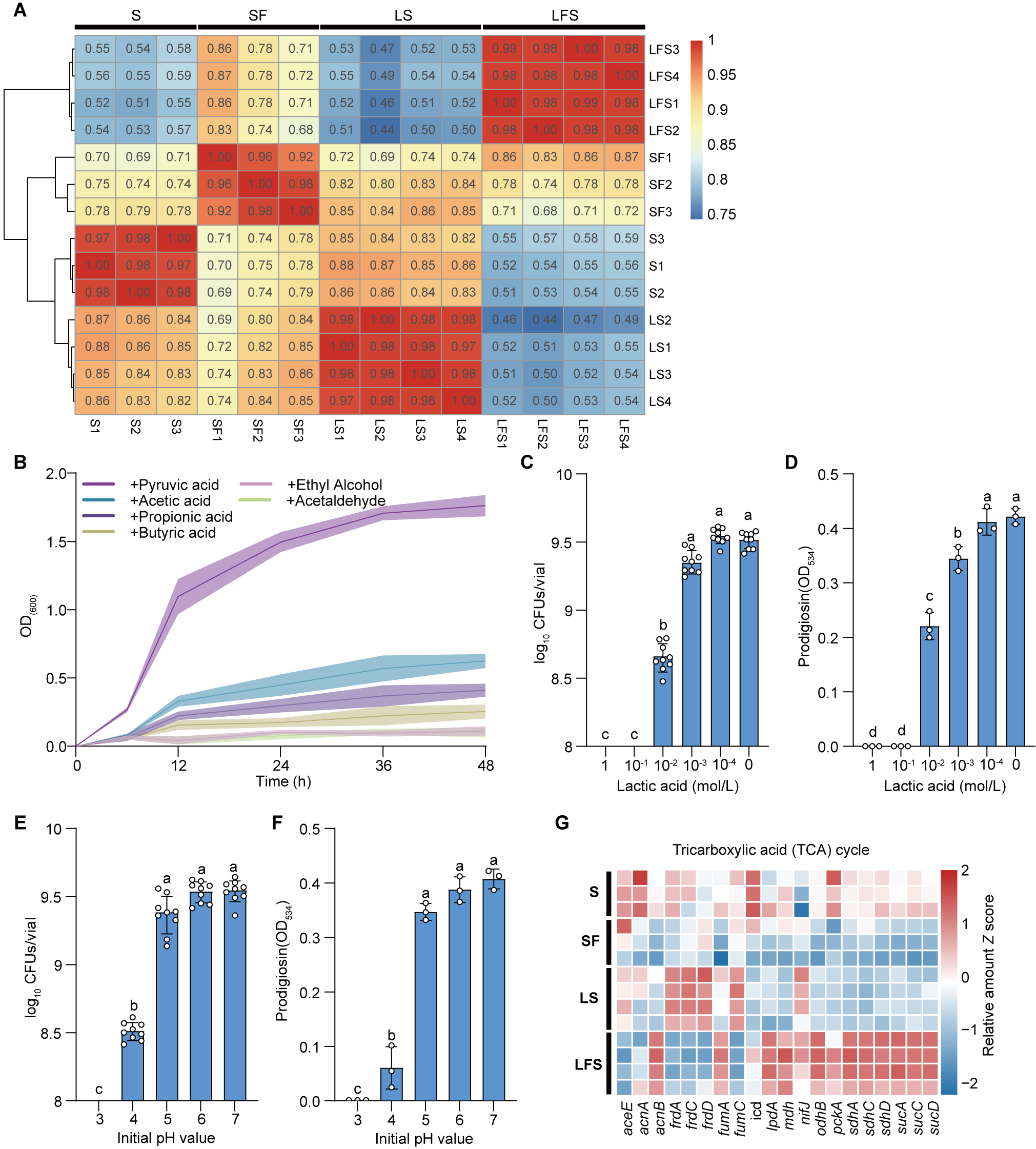
**

**Figure S3.** *Drosophila* larvae and *L. plantarum* independently enforced transcriptional profiling of *S. marcescens*. A) Gene expression similarity of *S. marcescens* in L, LF, LS and LFS groups. B) The growth curves of *S. marcescens* were monitored over time in a non-carbon CDM medium supplemented with individual carbon sources: pyruvic acid, ethyl alcohol, acetaldehyde, acetic acid, propionic acid, or butyric acid. For each condition, the OD values for bacterial growth and the lactate concentration were assessed separately over time (*n*  =  9). The solid line and shaded area of the curves represent the mean ± SD. C,D) *S. marcescens* bacterial load (C) and prodigiosin production content (D) at 24 hours with the addition of different concentrations of lactate to the medium (*n*  =  9). E,F) *S. marcescens* bacterial load (E) and prodigiosin production (F) at 24 hours with the addition of different concentrations of hydrochloric acid to adjust the environmental pH value (*n*  =  9). The data represent the means ± SD, and the different letters above the columns denote statistically significant differences (p < 0.05) between groups (one-way ANOVA followed by Tukey’s test for multiple comparisons) (C-F). G) Heatmap of the expression profiles of TCA cycle genes of *S. marcescens* in SF, LS, and LFS groups compared to in the S group. *Z* scores of the relative gene expression levels are displayed in the heatmaps (*n* = 4 independent experiments), with red representing higher and blue representing lower abundance.


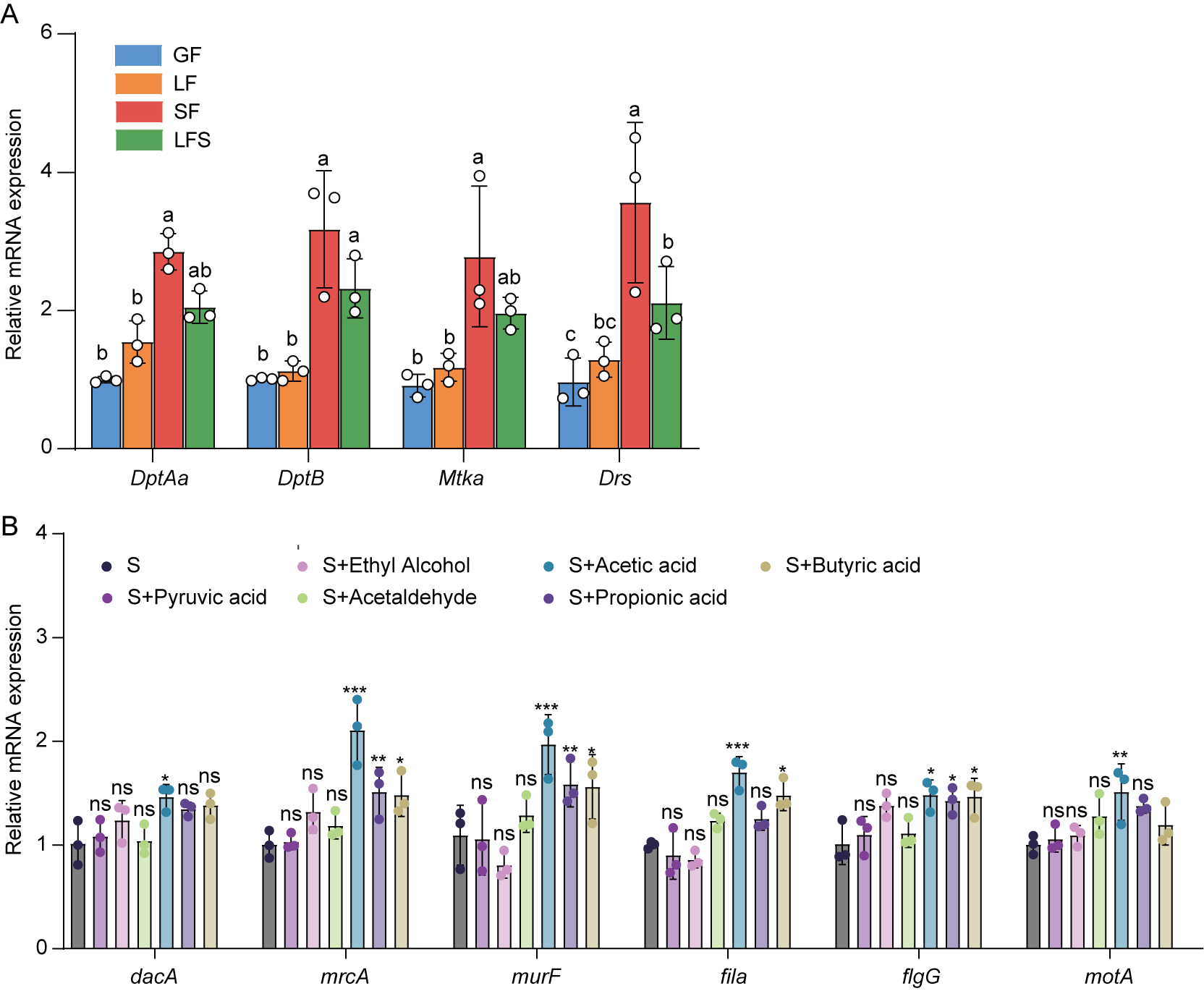


**Figure S4.** Expression levels of resistance genes in *S. marcescens* under various carbon source conditions. A) qRT-PCR was utilized to analyze the expression levels of antimicrobial peptides genes of larvae in after 24 h in GF, LF, SF, and LFS (*n*  =  3). The data represent the means ± SD. The different letters above the columns denote statistically significant differences (*p* < 0.05) between groups (one-way ANOVA followed by Tukey’s test for multiple comparisons). B) qRT-PCR was utilized to analyze the expression levels of resistance and virulence-associated genes of *S. marcescens* in fly diet with different carbon resource (*n* = 3). The data represent the means ± SD, and the significance analysis compared with the S group was performed by unpaired two-sided Student’s *t*-test. ns (non-significance) *p* >  0.05; **p* < 0.05; ***p*  <  0.01; ****p*  <  0.001.

**
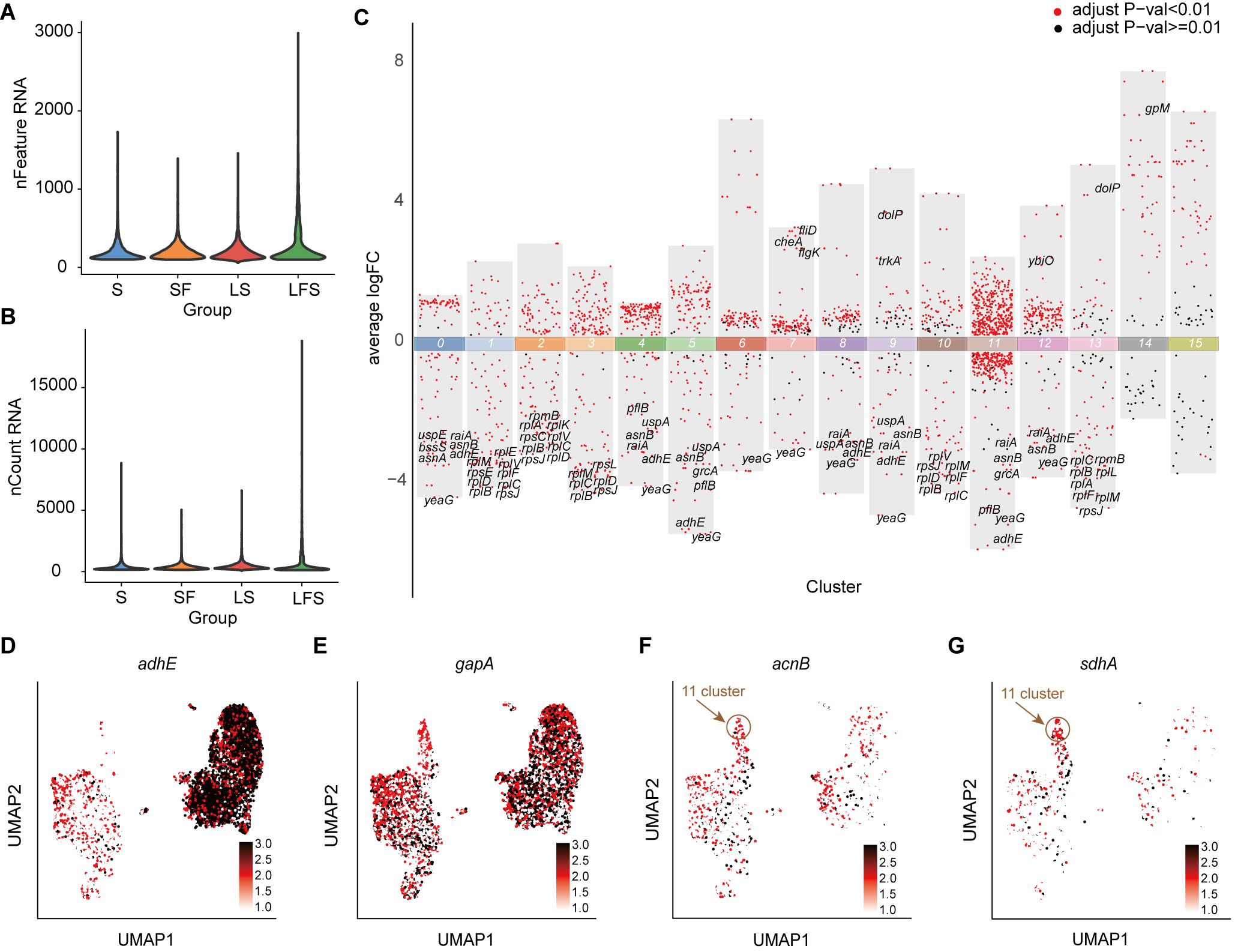
**

**Figure S5.** The phenotypic heterogeneity of *S. marcescens.* A,B) The nFeature RNA (A) and nCount RNA (B) for *S. marcescens* cells in L, LF, LS and LFS groups. C) Differential gene expression analysis showing upregulated and downregulated genes across all 15 clusters. An adjusted p value < 0.01 is indicated in red, while an adjusted p value ≥ 0.01 is indicated in black. D-G) The expression of *adhE* (D), *gapA* (E), *acnB* (F), *sdhA* (G) genes was highlighted on the UMAP.


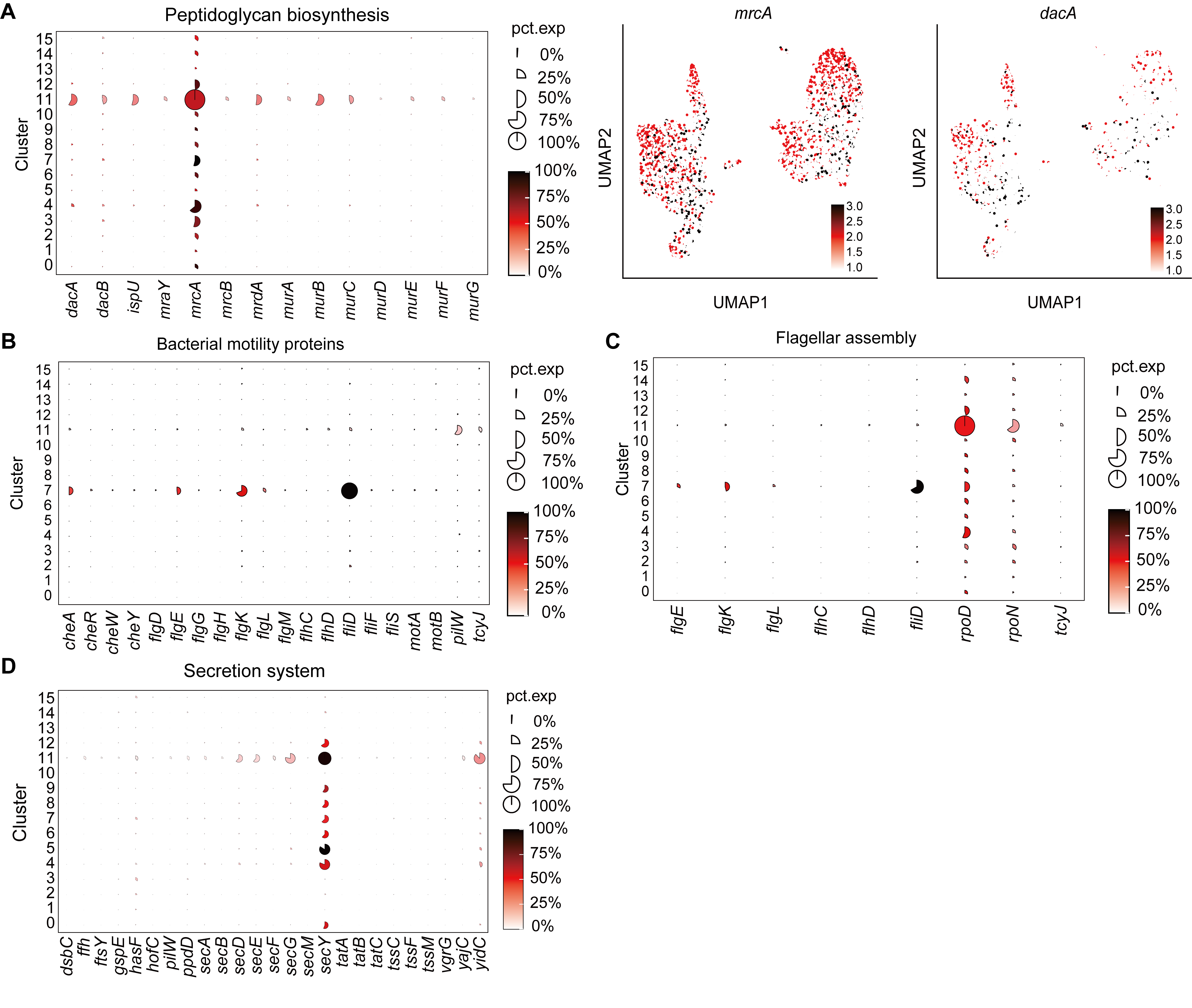


**Figure S6.** The heteroresistance of *S. marcescens.* A-D) The mean expression levels of pathways in different subclusters, overlays of expression of genes representative of each pathway on the UMAP. (A) peptidoglycan biosynthesis. (B) bacterial motility proteins. (C) flagellar assembly and (D) secretion system.


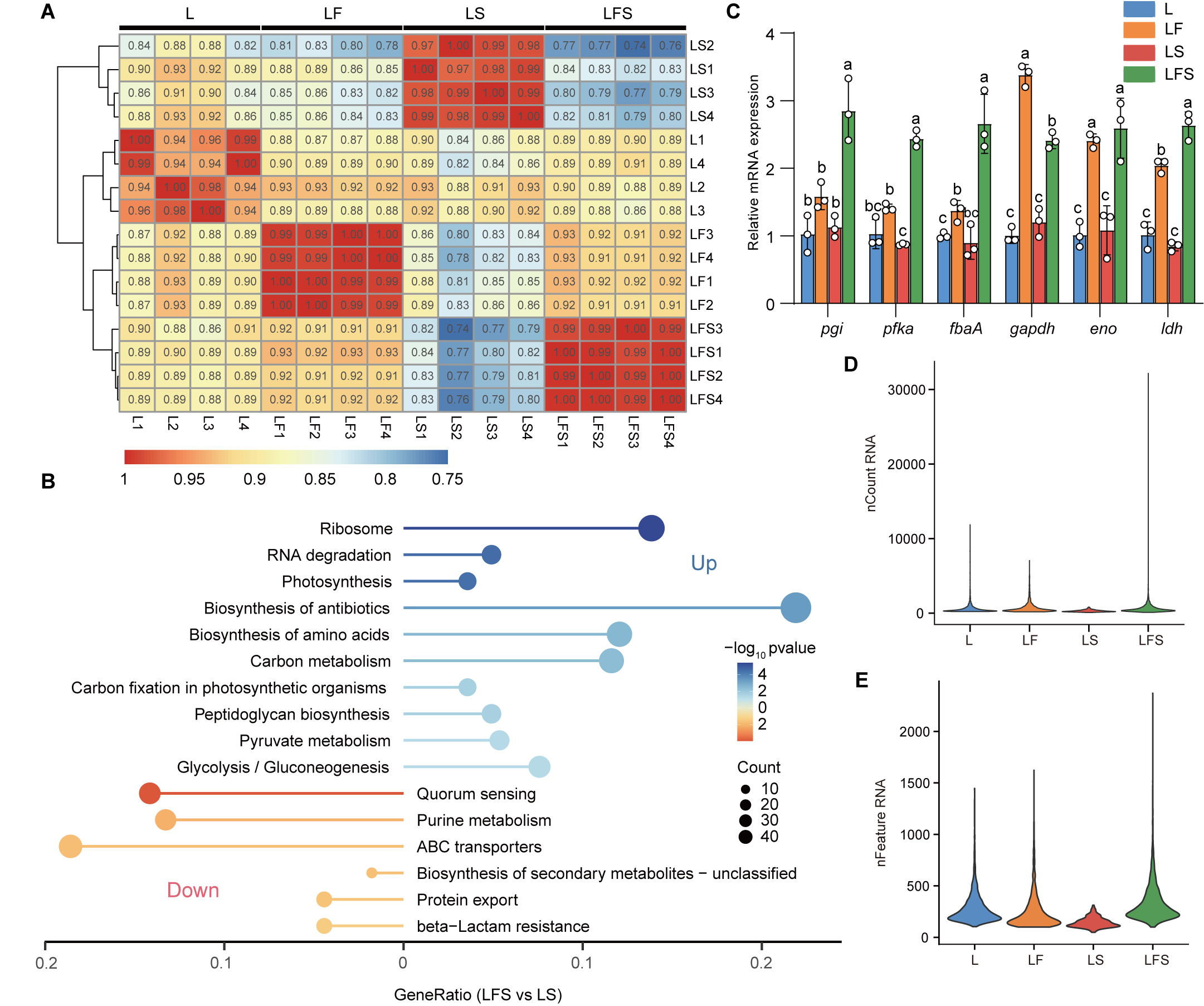


**Figure S7.** The lactic acid synthesis of *L. plantarum* under four treatment and single RNA sequencing quality control. A) Gene expression similarity between four group transcriptomes. B) KEGG enrichment analysis was conducted on genes significantly upregulated (log2fold > 1; p < 0.05) and downregulated (log2fold < -1; p < 0.05) in *L. plantarum* within the LFS vs LS. C) qRT-PCR was utilized to analyze the expression levels of glycolysis-associated genes in L, LF, LS and LFS groups. (*n* = 3). The data represent the means ± SD, and the different letters above the columns denote statistically significant differences (*p* < 0.05) between groups (one-way ANOVA followed by Tukey’s test for multiple comparisons). D,E) The nFeature RNA (D) and nCount RNA (E) for *L. plantarum* cells in L, LF, LS and LFS groups.

**Table S1. Features of the RT primers used in this work**

| Primer | Sequence | Lenght (bp) | GC% | Source |
| --- | --- | --- | --- | --- |
| 16S-RT-F | agagtttgatcctggctcag | 20 | 50 | This work |
| 16S-RT-R | ggttaccttgttacgactt | 20 | 42 | This work |
| pgi-RT-F | cggcggacgtttctctgtat | 20 | 55 | This work |
| pgi-RT-R | tttgcttgcatcagcagctc | 20 | 50 | This work |
| pfkA-RT-F | cgtgccgttgttcgtaaagg | 20 | 55 | This work |
| pfkA-RT-R | tgccacatcaagacgacgaa | 20 | 50 | This work |
| fbaA-RT-F | ttggttcaatcggcggagaa | 20 | 50 | This work |
| fbaA-RT-R | accgataccgcaagctaagt | 20 | 50 | This work |
| gapdh-RT-F | gacgtctagcattccgtcgt | 20 | 50 | This work |
| gapdh-RT-R | acgtccttgagttgtgtcgt | 20 | 55 | This work |
| eno-RT-F | acgtgacggagacaaagctc | 20 | 55 | This work |
| eno-RT-R | ggtcacgaacatcgtagcca | 20 | 55 | This work |
| ldh-RT-F | tgactctgctcgtttccgtc | 20 | 55 | This work |
| ldh-RT-R | tgcatgtgaccaaactggga | 20 | 50 | This work |
| dacA-RT-F | gcgtaagctgttgacgtgg | 19 | 57.9 | This work |
| dacA-RT-R | gtaccagaccggttcttccc | 20 | 60 | This work |
| mrcA-RT-F | aagcgttacggtgaaaacgc | 20 | 50 | This work |
| mrcA-RT-R | ttgttgcgtaccgactcctg | 20 | 55 | This work |
| murF-RT-F | aggtattgagcgctgaaccgat | 22 | 50 | This work |
| murF-RT-R | cgaaacgctcgcctttcagc | 20 | 60 | This work |
| flhA-RT-F | catgcgctgcgtcctttac | 19 | 57.9 | This work |
| flhA-RT-R | tcgaggtcatgcgaatctgg | 20 | 55 | This work |
| flgG-RT-F | actgacgttgacgacctttgt | 21 | 47.6 | This work |
| flgG-RT-R | accagagctttcggtttcct | 20 | 50 | This work |
| motA-RT-F | aacgccatggtcggtacttt | 20 | 50 | This work |
| motA-RT-R | gtaaccgttcaggctcgaca | 20 | 55 | This work |
| GFP-RT-F | actagtaagcttggtaccatgatggtgagcaagggcgagga | 41 | 51 | This work |
| GFP-RT-R | gatgatgatgatggtctctagaatcacttgtacagctcgtccatgc | 46 | 46 | This work |
| pgi-Promoter-F | caccgcatatgctggatcgcgcagcgggaaatgataac | 38 | 55 | This work |
| pgi-Promoter-R | ggaccatggtaccaagctgcctgagctgaagccaataa | 38 | 53 | This work |
| adhE-Promoter-F | caccgcatatgctggatcaatattctgctgctgcgcca | 38 | 53 | This work |
| adhE-Promoter-R | ggaccatggtaccaagctgtaaataagccgtgcgagcc | 38 | 55 | This work |
| sucA-Promoter-F | caccgcatatgctggatcgttacctgctgaacaaacac | 38 | 50 | This work |
| sucA-Promoter-R | ggaccatggtaccaagcttttcgccgtggttaacataa | 38 | 47 | This work |
| secG-Promoter-F | caccgcatatgctggatctctgcctgatgcacatgcag | 38 | 55 | This work |
| secG-Promoter-R | ggaccatggtaccaagcttagtacccgtatccttgcgg | 38 | 55 | This work |

**Table S2. Primer of *D. melanogaster* AMPs for qRT-PCR assay.**

| Symbol | Full gene name | Sequence (5′3′) | Size (bp) | GC (%) | Tm (°C) | Size (bp) | GC (%) |
| --- | --- | --- | --- | --- | --- | --- | --- |
|  |  |  |  |  |  |  |  |
| DptAa | *Diptericin A* | For. TTACTTTGCTGCGCAATCGCTT | 22 | 45 | 60.1 | 213 | 56.3 |
|  |  | Rev. TCCATATCCTCCATTCAGTCCA | 22 | 45 | 60.1 |  |  |
| *DptB* | *Diptericin B* | For. GTGCGTCGCCAGTTCCAATTG | 21 | 57 | 63.2 | 121 | 59.5 |
|  |  | Rev. CATCGAAGGAGTGGCGTCCAT | 21 | 57 | 63.2 |  |  |
| Mtka | *Metchnikowin* | For. GCGATTTTTCTGGCCCTGCTG | 21 | 57 | 63.2 | 115 | 58.3 |
|  |  | Rev. GGTTAGGATTGAAGGGCGACG | 21 | 57 | 63.2 |  |  |
| *Drs* | *Drosomycin* | For. GTACTTGTTCGCCCTCTTCGC | 21 | 57 | 63.2 | 138 | 60.9 |
|  |  | Rev. TCCTTGCACACACGACGACAG | 21 | 57 | 63.2 |  |  |

**Table S3. The quality of scRNA-seq datas.**

|  | L | S | LS | LF | FS | LFS |
| --- | --- | --- | --- | --- | --- | --- |
| RawData | 63.5G | 67.1G | 58.8G | 67.9G | 66G | 77.4G |
| Number of Reads | 211.7M | 223.5M | 196.1M | 226.4M | 220.1M | 258M |
| Sequencing Saturation% | 66.9 | 48.1 | 81.9 | 90.4 | 68.5 | 64.2 |
| Valid barcode % | 88.1 | 86.8 | 87.3 | 88.7 | 88.6 | 85.3 |
| Reads to Align% | 66.8 | 80.3 | 86.6 | 87.6 | 87.2 | 82 |
| Q30 bases in RNA read% | 93 | 92.05 | 90.85 | 92.98 | 90.32 | 93.7 |
| Total Genes Detected | 2687 | 4895 | 7409 | 2722 | 4896 | 7624 |
| Number of Valid Cells | 3056 | 7833 | 4412 | 1434 | 9360 | 3913 |
| UMI in Valid Cells | 1857438 | 2692723 | 1588818 | 911496 | 3352466 | 3309670 |
| Median Genes per Valid Cell | 234 | 151 | 141 | 161 | 167 | 349 |
| Median UMI Counts per Valid Cell | 398 | 246 | 279 | 381 | 293 | 559 |
